# A new *p*-terphenyl derivative from the insect-derived fungus *Aspergillus candidus* Bdf-2 and the synergistic effects of terphenyllin

**DOI:** 10.7717/peerj.8221

**Published:** 2020-01-02

**Authors:** Tijiang Shan, Yuyang Wang, Song Wang, Yunying Xie, Zehua Cui, Chunyin Wu, Jian Sun, Jun Wang, Ziling Mao

**Affiliations:** 1 Guangdong Key Laboratory for Innovative Development and Utilization of Forest Plant Germplasm, College of Forestry and Landscape Architecture, South China Agricultural University, Guangzhou, Guangdong, China; 2 Institute of Medicinal Biotechnology, Chinese Academy of Medical Sciences & Peaking Union Medical College, Beijing, China; 3 National Risk Assessment Laboratory for Antimicrobial Resistance of Animal Original Bacteria, South China Agricultural University, Guangzhou, Guangdong, China

**Keywords:** *p*-terphenyl derivatives, Insect-derived fungus, Secondary metabolites, Structure elucidation, Synergistic effects, *Aspergillus candidus* Bdf-2

## Abstract

A new *p*-terphenyl derivative 4″-deoxy-2′-methoxyterphenyllin (1), along with six known *p*-terphenyl derivatives (2–7), a known flavonoid derivative dechlorochlorflavonin (8) and a known fellutanine A (9), were isolated from the insect-derived strain of the fungus *Aspergillus candidus* Bdf-2, associated with *Blaptica dubia*. The structure of 1 was established by the analysis of the 1D and 2D NMR and HR-ESI-MS spectra. Compounds 1–9 were evaluated for antibacterial activities against *Staphylococcus aureus* ATCC29213, *Escherichia coli* ATCC25922 and *Ralstonia solanacearum*, and for antioxidant activities. Synergistic effects of compound 2 with the other compounds were also investigated. As a result, compound 6 displayed the best antibacterial activities in all single compound with MIC value of 32 µg/mL against *S. aureus* ATCC29213 and *R. solanacearum*, respectively. However, no antibacterial effect against *E. coli* ATCC25922 was detected from any single compound. The combination of 2 + 6 exhibited obvious synergistic effect against *S. aureus* ATCC29213 and the MIC value was 4 µg/mL. Compound 6 also showed the best antioxidant activity as a single compound with an IC_50_ value of 17.62 µg/mL. Combinations of 5 + 6, 2 + 4 + 5 and 2 + 4 + 5 + 6 displayed synergistic effect and their antioxidant activities were better than that of any single compound.

## Introduction

Cockroaches (Blattaria) are significant omnivores and have diverse hindgut microbiota encompassing hundreds of microbial species (Tinker & Ottesen, 2016). Furthermore, due to its unique niche, cockroach is likely to be the habitat for unique microorganism. In our previous study, five fungal isolates were isolated from the *Blaptica dubia*, and *Aspergillus candidus* Bdf-2 displayed the best antibacterial and antioxidant activities (Shan et al., 2019). Fungi belonging to the genus *Aspergillus* (Trichocomaceae) are one of the most prolific resources for compounds such as alkaloids (Si et al., 2018; Luo et al., 2019), steroids (Xing et al., 2019), anthraquinones (Xing et al., 2019; Li et al., 2019a), isocoumarins (Ma et al., 2019), polyketides (Qiao et al., 2018; Yin et al., 2018), terpenes (Li et al., 2019b), cyclopeptides (Liu et al., 2018), and *p*-terphenyls (Wang et al., 2017; Zhang et al., 2018). Metabolites isolated from species of the genus *Aspergillus* have continually attracted the interest of pharmacologists due to their broad array of biological activities and their structural diversity (Wang et al., 2017). *p*-Terphenyl derivatives have been reported with diverse biological activities, such as neuraminidase inhibitory (Guo et al., 2012), *α*-glucosidase inhibitory (Zhang et al., 2018), antioxidant (Yen et al., 2001), cytotoxic (Cai et al., 2011; Sangsopha et al., 2019; Yurchenko et al., 2017), antibacterial (Yan et al., 2017), and immunosuppressive activities (Ivanets et al., 2018).

**Figure 1:**
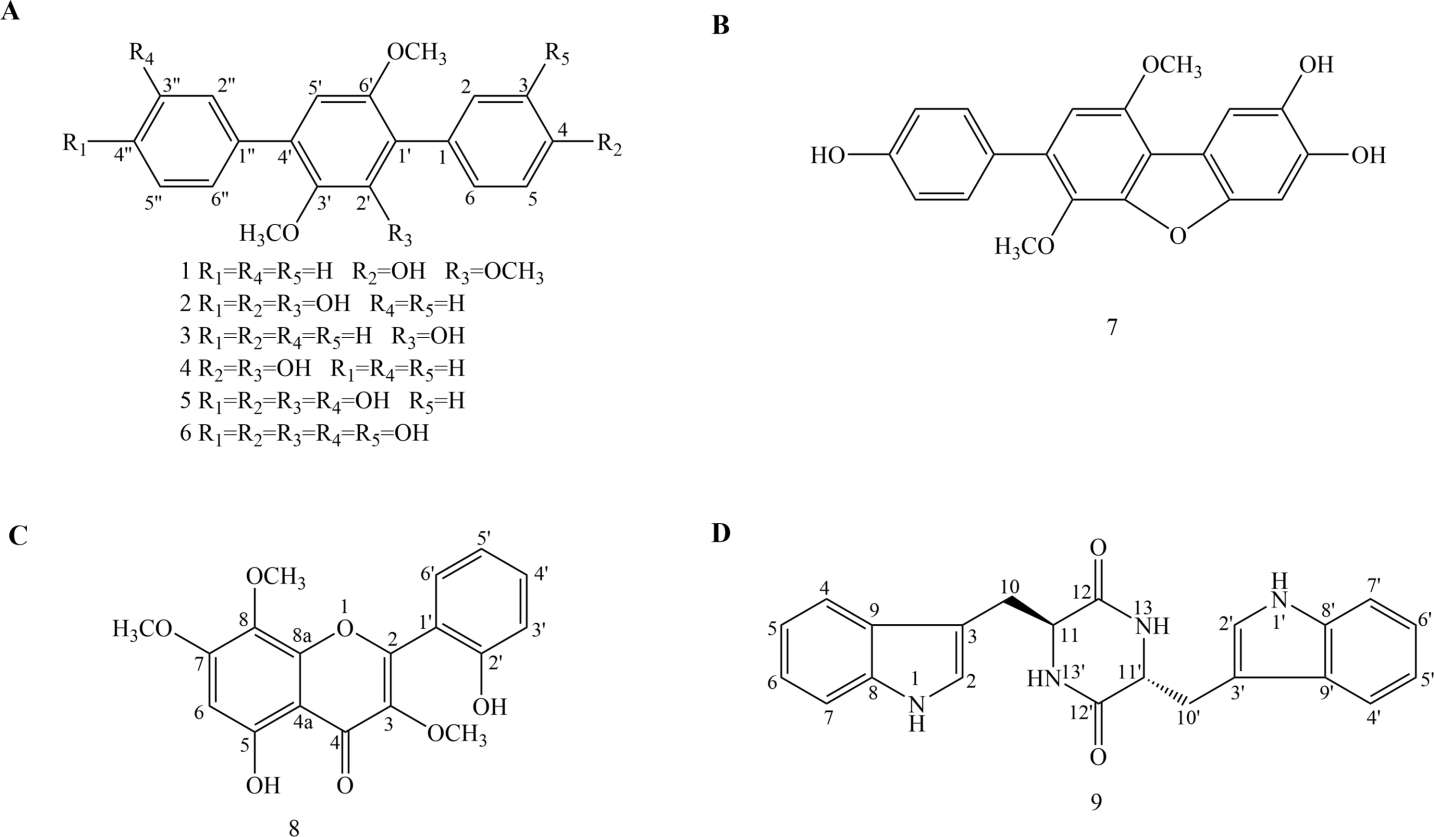
Chemical structures of compounds 1–9. (A) Chemical structures of compounds 1–6. (B) Chemical structure of compound 7. (C) Chemical structure of compound 8. (D) Chemical structure of compound 9.

The fungus *Aspergillus candidus* Bdf-2 was isolated from the abdomen of *Blaptica dubia*. Although the genus *Aspergillus* have been proven to be a large reservoir of *p*-terphenyl derivatives and other biologically active compounds, not many insect-derived fungal strains of the *Aspergillus candidus* have been described. We wondered whether cockroaches which can survive in a specific environment, were related to the symbiotic microorganisms living in their bodies. Based on promising screening results in search of producers of biologically active compounds, the insect-derived fungus *Aspergillus candidus* Bdf-2 was selected for further studies. Here we deal with the description, isolation and characterization of a new *p*-terphenyl derivative (**1**) together with other eight previously known compounds **2**–**9** (Fig. 1). All compounds (**1**–**9**) were evaluated for their antibacterial and antioxidant activities. The details of isolation, structure determination and biological activities of these compounds are presented herein.

## Materials & Methods

### General experimental procedures

The UV spectra were scanned on a UV-2600 instrument (Shimadzu, Kyoto, Japan). IR spectra were recorded on a Shimadzu Affinity-1 instrument (Shimadzu, Kyoto, Japan). HRESIMS spectra were obtained on a Q-TOF mass spectrometer from Bruker maXis, with an electrospray ionization (ESI) interface (Bruker, Fremont, CA, USA). Standard 1D and 2D NMR spectra were recorded on a Bruker Avance-600 NMR spectrometer (^1^H at 600 MHz and ^13^C at 151 MHz) (Bruker, Fremont, CA, USA). Silica gel (200–300 mesh, Qingdao Marine Chemical Inc., Qingdao, China), and Sephadex LH-20 (GE Healthcare, Uppsala, Sweden) were used for column chromatography. Semi-preparative HPLC separations were carried out on a semi-preparative HPLC instrument equipped with a UC-3281 pump and a UC-3292S UV detector, using an XB-C_18_ column (250 mm × 10 mm, 10 µm, Welch, Shanghai, China). Precoated silica gel GF-254 plates (Qingdao Marine Chemical Inc., Qingdao, China) were used for analytical TLC and Spots were visualized under UV light (254 or 365 nm).

### Fungal material

The fungus *Aspergillus candidus* Bdf-2 (Fig. S1) was isolated from the abdomen of *Blaptica dubia* which were purchased from Lianwanjia Dubia exclusive store in Qingdao on November 30, 2016. The isolate was identified as *Aspergillus candidus* by analysis of its morphological characteristics and the rDNA gene internal transcribed spacer (ITS) sequence (GenBank accession number MH681592) (Shan et al., 2019), as well as beta-tubulin (tub2) gene sequence (GenBank accession number MN533958), RNA polymerase II second largest subunit (RPB2) gene sequence (GenBank accession number MN533959) and large subunit ribosomal RNA gene sequence (GenBank accession number MN533960) (Figs. S2–S5). The fungus was stored on potato dextrose agar (PDA) slants at 4 °C in the College of Forestry and Landscape Architecture, South China Agricultural University, Guangzhou, China. The culture was also stored at China Center for Type Culture Collection (CCTCC) and the strain preservation number was CCTCC AF 2019013.

### Fermentation, extraction, and isolation

The fungus *Aspergillus candidus* Bdf-2 was cultured on potato dextrose agar (PDA) (potato 200 g/L, dextrose 20 g/L, and agar 20 g/L) medium in Petri dishes at 28 °C for 5 days. Then, three agar plugs (0.5 × 0.5 cm) were inoculated in a 500 mL Erlenmeyer flask containing 200 mL of potato dextrose broth (PDB) (potato 200 g/L and dextrose 20 g/L) medium and incubated on a rotary shaker at 150 rpm and 28 °C for 5 days. The obtained liquid seeds were added into the sterilized rice culture medium (6.0 kg) in the incubators at 28 °C for 60 days before harvest. The leavening was extracted with ethyl acetate (EtOAc). Then, the EtOAc extracts were dissolved in 1 L methanol (MeOH) and extracted with petroleum ether at room temperature. The petroleum ether and MeOH extracts were dried using vacuum concentration to give crude extracts of 20.7 g and 113.5 g, respectively.

The petroleum ether crude extract (20.7 g) was subjected to column chromatography over silica gel (200–300 mesh) eluted with a gradient of petroleum ether-acetone (100:0–0:100) to give three fractions (Fr. A-C). Fraction A (5.013 g) was chromatographed over Sephadex LH-20 (eluted with CHCl_3_-MeOH, 1:1) to give 24 further subfractions, A_1_ to A_24_, and subfractions A_8_–A_12_ were combined and further purified by recrystallization to afford 4, 4″-deoxyterphenyllin (**3**) (19.9 mg). For the conditions of recrystallizations, the impure compound was first eluted with insoluble solvents such as petroleum ether or ethyl acetate at room temperature, followed by dissolution with soluble acetone or methanol, and repeated the above steps after crystallization until the compound was purified. Fraction B (4.072 g) was also chromatographed over Sephadex LH-20 (eluted with CHCl_3_-MeOH, 1:1) to give 77 further subfractions, B_1_ to B_77_. Subfractions B_42_–B_56_ were combined and further purified by semi-preparative HPLC (eluted with MeOH–H_2_O over 35 mins, 70:30, with an isocratic 0.01% TFA modifier, 4 mL/min, 210 nm) to afford dechlorochlorflavonin (**8**) (9.3 mg) and 4″-deoxy-2′-methoxyterphenyllin (**1**) (8.6 mg).

The MeOH extracts (113.5 g) were first subjected to column chromatography over silica gel (200–300 mesh) eluted with a gradient of petroleum ether-acetone (100:0–0:100) to give four fractions (Fr. D–G). Fraction D (62.4 g) was purified by recrystallization to afford terphenyllin (**2**) (43.5 g) and the residue was further chromatographed over Sephadex LH-20 (eluted with CHCl_3_-MeOH, 1:1) to give 18 subfractions. Subfractions D_6_-D_12_ were combined and further purified by recrystallization to afford candidusin A (**7**) (61.5 mg). Fraction E (435.6 mg) was first recrystallized and then further purified by semi-preparative HPLC (eluted with MeOH–H_2_O over 35 mins, 70:30, with an isocratic 0.01% TFA modifier, 4 mL/min, 210 nm) to afford 4″-deoxyterphenyllin (**4**) (24.7 mg). Fraction G (2.1 g) was chromatographed over Sephadex LH-20 (eluted with CHCl_3_-MeOH, 1:1) to give 24 further subfractions. Subfractions G_6_-G_9_ were combined and further recrystallized to afford fellutanine A (**9**) (14.5 mg). Subfractions G_11_-G_15_ were combined and further recrystallized to afford 3″-hydroxyterphenyllin (**5**) (65.4 mg). Subfractions G_18_-G_20_ was purified by semi-preparative HPLC (eluted with MeOH–H_2_O over 35 mins, 53:47, with constant 0.01% TFA modifier, 4 mL/min, 210 nm) to obtain 3, 3″-dihydroxyterphenyllin (**6**) (54.8 mg).

### Compound identification

**Figure 2:**
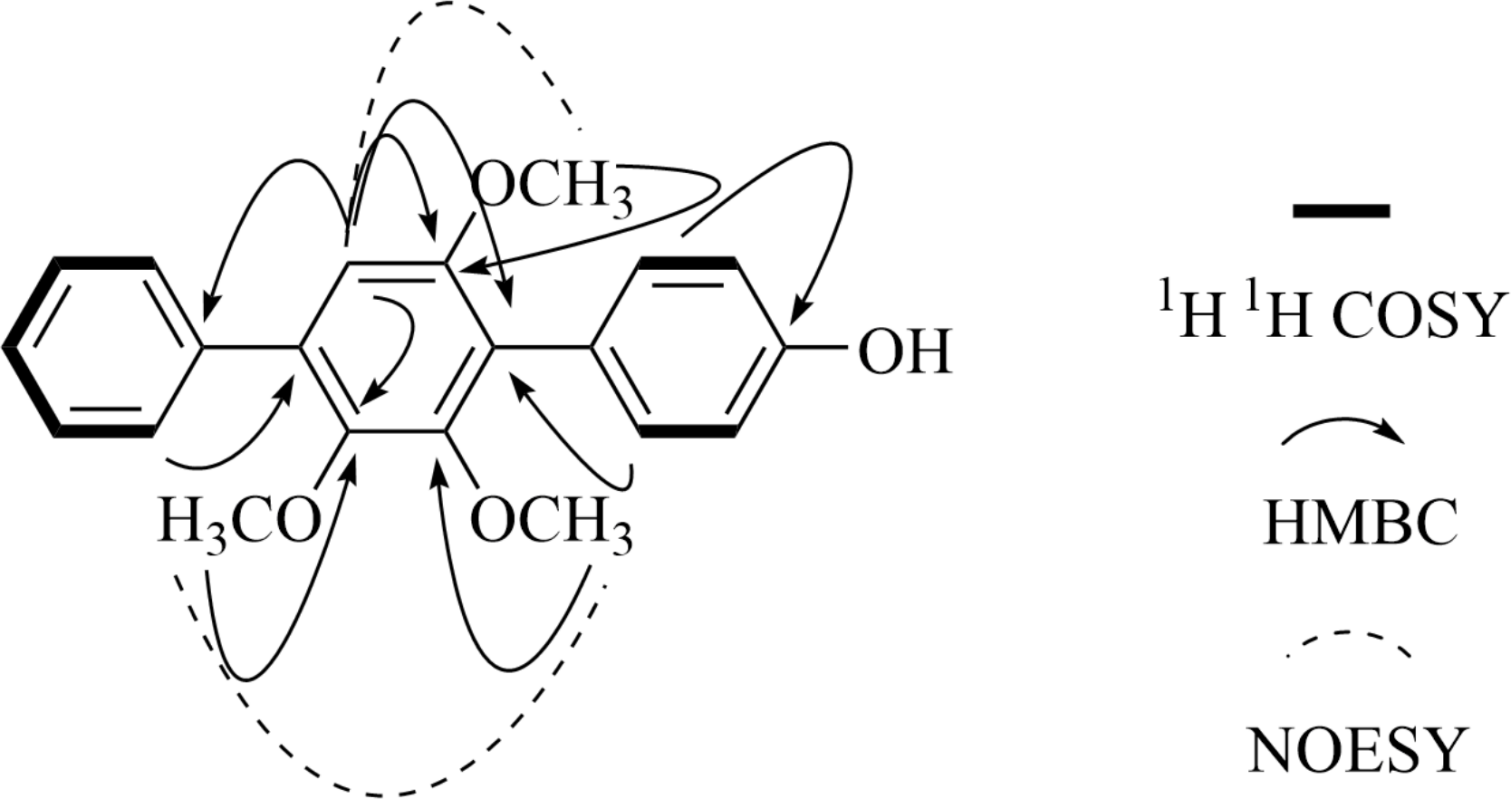
Key 2D NMR data of 4″-Ddeoxy- 2′-methoxyterphenyllin (1).

4″-Deoxy-2′-methoxyterphenyllin (**1**): light yellow powder; UV (MeOH) *λ*_max_ 213, 266 nm; IR *ν*_max_ 3354, 2959, 2932, 1591, 1520, 1474, 1458, 1435, 1389, 1341, 1265, 1227, 1103, 1080, 1015, 997, 837, 802, 773, 702, 652 cm^−1^; HR-ESI-MS *m*/*z* 337.1439 [M + H]^+^ (calcd for C_21_H_21_O_4_, 337.1434), 359.1261 [M + Na]^+^ (calcd for C_21_H_20_O_4_Na, 359.1254), ^13^C NMR and ^1^H NMR see Tables 1 and 2, and the key 2D NMR data see Fig. 2.

**Table 1:** ^13^C-NMR Data of compounds 1–7 (*δ* in ppm, 1, 4 in acetone-*d*_6_, 2 in DMSO-*d*_6_, 3 in CDCl_3_, 5–7 in CD_3_OH).

**Position**	**1**	**2**	**3**	**4**	**5**	**6**	**7**
1	125.92	124.56	133.41	125.93	125.58	127.05	106.23
2	132.74	131.84	130.93	133.04	129.72	116.39	108.68
3	115.36	114.34	128.70	115.33	114.79	146.10	143.34
4	157.30	155.90	127.51	157.12	156.45	145.93	146.74
5	115.36	114.34	128.70	115.33	114.79	117.28	99.09
6	132.74	131.84	130.93	133.04	129.72	121.67	151.72
1′	125.99	116.99	117.27	118.59	117.48	118.88	116.00
2′	153.02	148.12	147.40	149.19	147.78	149.22	150.48
2′-OMe	61.04	55.61	–	–	–	–	–
3′	145.87	139.34	139.09	139.57	139.37	140.82	137.62
3′-OMe	60.84	55.61	61.12	56.25	59.46	56.59	56.33
4′	135.40	132.35	133.20	133.61	132.94	134.45	132.59
5′	109.07	103.02	104.28	104.51	103.62	105.09	106.55
6′	154.40	153.09	153.65	154.71	153.53	154.91	151.44
6′-OMe	56.43	60.05	56.22	60.99	55.14	60.90	61.38
1″	139.58	128.78	138.28	128.14	129.70	131.76	131.27
2″	130.11	129.72	128.97	129.73	114.48	115.88	131.73
3″	129.05	115.19	128.70	129.25	144.14	145.58	116.08
4″	128.05	156.72	127.75	140.40	143.77	145.21	157.72
5″	129.05	115.19	128.70	129.25	118.05	119.49	116.08
6″	130.11	129.72	128.97	129.73	122.47	123.88	131.73

**Table 2:** ^1^H-NMR Data for compounds 1–7 (*δ* in ppm, *J* in Hz, 1, 4 in acetone-*d*_6_, 2 in DMSO-*d*_6_, 3 in CDCl_3_, 5–7 in CD_3_OH).

**Position**	**1**	**2**	**3**	**4**	**5**	**6**	**7**
1	–		–	–	–	–	–
2	7.22(d,8.4)	7.11(d,7.8)	7.46∼7.51(m)	7.26(d,8.4)	7.44(d,8.4)	6.84(s)	7.43(s)
3	6.89(d,8.4)	6.77(d,8.4)	7.46∼7.51(m)	6.87(d,8.4)	6.85(m)	–	–
3-OH	–	–	–	–	–	–	–
4	–	–	7.40(m)	–	–	–	–
4-OH	–	9.31(s)	–	–	–	–	–
5	6.89(d,8.4)	6.77(d,8.4)	7.46∼7.51(m)	6.87(d,8.4)	6.85(m)	6.81(d,6.6)	7.01(s)
6	7.22(d,8.4)	7.11(d,7.8)	7.46∼7.51(m)	7.26(d,8.4)	7.44(d,8.4)	7.12(s)	–
1′	–	–	–	–	–	–	–
2′	–	–	–	–	–	–	–
2′-OH		8.51(s)	5.97(s)	–	–	–	–
2′-OMe	3.58(s)	–	–	–	–	–	–
3′	–	–	–	–	–	–	–
3′-OMe	3.57(s)	3.31(s)	3.47(s)	3.37(s)	3.37(s)	3.42(s)	3.76(s)
4′	–	–	–	–	–	–	–
5′	6.77(s)	6.40(s)	6.52(s)	6.52(s)	6.41(s)	6.43(s)	6.60(s)
6′	–	–	–	–	–	–	–
6′-OMe	3.73(s)	3.64(s)	3.76(s)	3.72(s)	3.63(s)	3.67(s)	3.92(s)
1″	–	–	–	–	–	–	–
2″	7.61(d, 7.3)	7.44(d,7.8)	7.68(m)	7.65(d,7.8)	6.85(m)	6.84(s)	7.38(d,8.4)
3″	7.45(t, 7.7)	6.85(d,7.8)	7.46∼7.51(m)	7.45(t,7.8)	–	–	6.85(d,9.0)
3″-OH	–	–	–	–	–	–	–
4″	7.36(t, 7.4)	–	7.40(m)	7.37(m)	–	–	–
4″-OH	–	9.52(s)	–	–	–	–	–
5″	7.45(t, 7.7)	6.85(d,7.8)	7.46∼7.51(m)	7.45(t,7.8)	6.71(dd,8.4,2.4)	6.72(d,5.4)	6.85(d,9.0)
6″	7.61(d, 7.3)	7.44(d,7.8)	7.68(m)	7.65(d,7.8)	6.81(m)	6.96(d,5.4)	7.38(d,8.4)

Terphenyllin (**2**): white crystal; HR-ESI-MS *m*/*z* 339.1233 [M + H]^+^ (calcd for C_20_H_19_O_5_, 339.1227), 361.1048 [M + Na]^+^ (calcd for C_20_H_18_O_5_Na, 361.1046). ^13^C NMR and ^1^H NMR see Tables 1 and 2 and the data were consistent with the literature (Kurobane et al., 1979).

4, 4″-Deoxyterphenyllin (**3**): white powder; HR-ESI-MS *m*/*z* 307.1335 [M + H]^+^ (calcd for C_20_H_19_O_3_, 307.1329), 329.1152 [M + Na]^+^ (calcd for C_23_H_26_O_7_Na, 329.1148), ^13^C NMR and ^1^H NMR see Tables 1 and 2 and the structure was confirmed by comparison with literature data (Biggins et al., 2011).

4″-Deoxyterphenyllin (**4**): white crystal; HR-ESI-MS *m*/*z* 323.1274 [M + H]^+^ (calcd for C_20_H_19_O_4_, 323.1278), 345.1096 [M + Na]^+^ (calcd for C_20_H_18_O_4_Na, 345.1097), ^13^C NMR and ^1^H NMR see Tables 1 and 2 and the data were consistent with the literature (Takahashi et al., 1976).

3″-Hydroxyterphenyllin (**5**): white powder; HR-ESI-MS *m*/*z* 355.1180 [M + H]^+^ (calcd for C_20_H_19_O_6_, 355.1176), 377.0999 [M + Na]^+^ (calcd for C_20_H_18_O_6_Na, 377.0996), ^13^C NMR and ^1^H NMR see Tables 1 and 2 and the data were consistent with the literature (Ivanets et al., 2018).

3, 3″-Dihydroxyterphenyllin (**6**): light yellow solid; HR-ESI-MS *m*/*z* 371.1127 [M + H]^+^ (calcd for C_20_H_19_O_7_, 371.1125), 393.0945 [M + Na]^+^ (calcd for C_20_H_18_O_7_Na, 393.0945), ^13^C NMR and ^1^H NMR see Tables 1 and 2 and the structure was confirmed by comparison with literature data (Kobayashi et al., 1985).

Candidusin A (**7**): gray amorphous solid; HR-ESI-MS *m*/*z* 353.1024 [M + H]^+^ (calcd for C_20_H_17_O_6_, 353.1020), 375.0844 [M + Na]^+^ (calcd for C_20_H_16_O_6_Na, 375.0839), ^13^C NMR and ^1^H NMR see Tables 1 and 2 and the data were consistent with the literature (Liu et al., 2012).

Dechlorochlorflavonin (**8**): yellow powder; HR-ESI-MS *m*/*z* 353.1024 [M + H]^+^ (calcd for C_20_H_17_O_6_, 353.1020), 375.0844 [M + Na]^+^ (calcd for C_20_H_16_O_6_Na, 375.0839). ^1^H-NMR (600 MHz, CDCl_3_) *δ* 7.93 (1H, m, H-6′), 7.80 (1H, m, H-4′), 7.14 (1H, m, H-3′), 7.14 (1H, m, H-5′), 6.46 (1H, s, H-6), 3.96 (3H, s, 7-OMe), 3.89 (3H, s, 3-OMe), 3.88 (3H, s, 8-OMe); ^13^C-NMR (151 MHz, CDCl_3_) *δ* 178.02 (C-4), 159.12 (C-7), 157.66 (C-5), 156.09 (C-2), 155.72 (C-2′), 149.25 (C-8a), 137.51 (C-3), 133.69 (C-4′), 129.86 (C-5′), 129.31 (C-8), 121.22 (C-6′), 119.99 (C-3′), 118.36 (C-1′), 105.42 (C-4a), 96.06 (C-6), 62.44 (3-OMe), 61.91 (8-OMe), 56.65 (7-OMe) (Watanabe et al., 2001).

Fellutanine A (**9**): pink solid; HR-ESI-MS *m*/*z* 373.1652 [M + H]^+^ (calcd for C_22_H_21_N_4_O_2_, 373.1659), 395.1473 [M + Na]^+^ (calcd for C_22_H_21_N_4_O_2_Na, 395.1478), ^1^H-NMR (600 MHz, CDCl_3_) *δ* 7.45 (d, 8.4, 2H, H-7, H-7′), 7.31 (d, 8.4, 2H, H-4, H-4′), 7.09 (m, 2H, H-5, H-5′), 7.01 (m, 2H, H-6, H-6′), 6.47 (s, 2H, H-2, H-2′), 4.04 (m, 2H, H-11, H-1 1′), 2.93 (m, 2H, H-10a, H-10a′), 2.18 (m, 2H, H-10b, H-10b′); ^13^C-NMR (151 MHz, CDCl_3_) *δ* 169.85 (C-12, C-1 2′), 138.19 (C-8, C-8′), 128.75 (C-9, C-9′), 126.05 (C-2, C-2′), 122.67 (C-5, C-5′), 120.22 (C-6, C-6′), 119.82 (C-7, C-7′), 112.56 (C-4, C-4′), 109.60 (C-3, C-3′), 57.00 (C-11, C-11′), 31.52 (C-10, C-10′) (Kozlovsky et al., 2000).

### Antibacterial assay

One Gram-positive (*S. aureus* ATCC29213) and two Gram-negative (*E.* coli ATCC25922 and *R. solanacearum*) bacteria were selected for antibacterial activity assay. Amikacin was used as a positive control. The minimum inhibitory concentrations (MIC) of the compounds and positive control were determined in sterile 96-well plates using the microculture tetrazolium (MTT) assay as described previously (Ouyang et al., 2018; Zhao et al., 2010). The synergistic antibacterial effects of terphenyllin (**2**) with different compounds were determined by the same method. The compounds in different combinations were mixed together at the same concentration and volume.

### Antioxidant assay

Radical scavenging assay was determined by a microplate spectrophotometric method based on the reduction of a methanol solution of DPPH according to our previous reports (Ouyang et al., 2018). Inhibition (%) of free radical (DPPH) in percent was determined as [(*A*_control_ − *A*_sample_)∕*A*_control_] × 100, where *A*_control_ is the absorbance of the control reaction containing all reagents except the test sample, and *A*_sample_ is the absorbance of the test compounds. Tests were carried out in triplicate. BHT was used as a positive control. The IC_50_ value was calculated using linear relation between the compound concentration and probability of the percentage of DPPH inhibition. The synergistic antioxidant effects of terphenyllin (**2**) with different compounds were determined by the same method. The compounds in different combinations were mixed together at the same concentration and volume as well as the synergistic antibacterial effects assay.

## Results

### Purification and characterization

The solid fermentation product of *Aspergillus candidus* Bdf-2 was extracted with ethyl acetate (EtOAc) and the resulting extracts were partitioned into petroleum ether- and methanol (MeOH)-soluble fractions. The petroleum ether and MeOH fractions were further purified by conventional chromatographic techniques. One new *p*-terphenyl derivative (**1**) and eight known compounds (**2**–**9**) were structurally characterized (Fig. 1). Among them, compounds **1**–**7** were *p*-terphenyl derivatives. Interestingly, terphenyllin (**2**) (43.5 g/113.5 g) was the dominant compound in the EtOAc crude extracts of *Aspergillus* sp. Bdf-2.

### Structure elucidation

The chemical structure of the new compound was elucidated by UV, IR, HR-ESI-MS and 1D and 2D NMR experiments (COSY, NOESY, HSQC and HMBC) (Figs. S6–S14). Compound **1** was obtained as a light yellow powder. Its molecular formula was established as C_21_H_20_O_4_ (twelve degrees of unsaturation), according to the prominent pseudomolecular ion peak at *m*/*z* 337.1439 [M + H]^+^ and 359.1261 [M + Na]^+^ in the HR-ESI-MS spectrum (Fig. S8). The IR spectrum exhibited the absorption bands due to the presence of hydroxyl (3354 cm^−1^), and aromatic (1591 and 1520 cm^−1^) moieties. The ^13^C-NMR (Table 1) and HSQC spectra suggested 21 carbon signals, including four *O*-substituted aromatic quaternary carbons (*δ*_C_ 157.30, 154.40, 153.02 and 145.87), ten methines and three methoxy carbons (*δ*_C_ 61.04, 60.84 and 56.43). The ^1^H-NMR spectrum (Table 2) of **1** showed the presence of one monosubstituted benzene at *δ*_H_ 7.61 (2H, t, 7.3, H-2″, 6″), 7.55 (2H, t, 7.7, H-3″, 5″) and 7.36(1H, t, 7.4, H-4″). The existence of a para-substituted benzene ring was also suggested by the ^1^H NMR data of *δ*_H_ 7.22 (2H, d, 8.4, H-2, 6) and 6.89 (2H, d, 8.4, H-3, 5). Therefore, an aromatic proton of pentasubstituted benzene at *δ*_H_ 6.77 (1H, s, H-5′) was identified based on the left 4 degrees of unsaturation and the ^13^C NMR. The NMR spectral data of **1** indicated the presence of the *p*-terphenyl skeleton with one hydroxyl and three methoxy groups.

The HMBC correlations (Fig. 2) of 2′-OCH_3_ with C-2′, 3′-OCH_3_ with C-3′, 6′-OCH_3_ with C-6′, and H-5′ with C-1′, C-3′, C-4′, and C-6′ and the NOE between 3′-OCH_3_ and 4′-OCH_3_, H-5′ to 6′-OCH_3_ suggested that the structure of the pentasubstituted benzene ring was 2′, 3′ and 6′ three methoxyl substituted. So the hydroxyl group was set at the para-substituted benzene ring unit. The HMBC signals of H-5′ with C-1″, H-2″ with C-4′, H-2 with C-1′ established the structure of compound **1**, and which was named 4″-dehydroxy-2′-methoxyterphenyllin.

The known compounds were identified according to their spectroscopic data (^1^H NMR, ^13^C NMR and HR-ESI-MS) as well as comparison with literature data. The clear HR-ESI-MS and NMR spectra of known compounds were also provided in Supplementary Materials to further confirm the structure (Figs. S15–S38).

### Antibacterial assay

All isolated compounds were evaluated for the antibacterial activities to a Gram-positive bacterium (*S. aureus* ATCC29213) and two Gram-negative bacteria (*E. coli* ATCC25922 and *R. solanacearum*), and the results were shown in Table 3. Amikacin was used as a positive control for antibacterial activity and the MIC values were all 1 µg/mL. This was the first time that compounds **1–9** were studied for antibacterial activities against *R. solanacearum.* Compounds **5**, **6** and **7** showed antibacterial activity against *S. aureus* ATCC29213 and *R. solanacearum*. Among them, 3, 3″-dihydroxyterphenillin (**6**) displayed the best antibacterial activity with MIC values of 32 µg/mL against *S. aureus* ATCC29213 and *R. solanacearum*, respectively. Compounds **5** and **7** demonstrated the equal inhibitory activity against *S. aureus* ATCC29213 and *R. solanacearum* with MIC values of 64 µg/mL. However, none of the nine compounds showed inhibitory activity against *E. coli* ATCC25922 when applied individually. Surprisingly, when *E.* coli ATCC25922 was treated with the combinations of **2** + **5** and **2** + **7,** significant inhibition activity was observed, and the MIC values were 128 µg/mL and 256 µg/mL, respectively. Compounds **5** and **6** also showed a synergistic effect against *E. coli* ATCC25922 when combined and the MIC value was 256 µg/mL. The combination of **2** + **6** displayed the strongest inhibitory activity against *S. aureus* ATCC29213 among the tested samples and the MIC value was 4 µg/mL, which demonstrated an obvious synergistic antibacterial effect. Combinations of **2** + **7**, **5** + **6**, **2** + **5**, **2** + **1** and **2** + **4** also showed synergistic antibacterial effect against *S. aureus* ATCC29213, which was significantly stronger than any single compound. For *R. solanacearum*, only combinations of **2** + **1** and **2** + **4** showed synergistic antibacterial effect.

**Table 3:** Antibacterial activities.

**Compound**	**MIC (µg/mL)**			**Combination**	**MIC (µg/mL)**		
	***E. coli*ATCC25922**	***S. aureus*ATCC29213**	***R. solanacearum***		***E. coli*ATCC25922**	***S. aureus*ATCC29213**	***R. solanacearum***
**1**	–	–	–	**2 + 1**	–	64	64
**2**	–	–	–	**2 + 3**	–	–	–
**3**	–	–	–	**2 + 4**	–	128	256
**4**	–	–	–	**2 + 5**	128	32	64
**5**	–	64	64	**2 + 6**	–	4	128
**6**	–	32	32	**2 + 7**	256	16	64
**7**	–	64	64	**5 + 6**	256	16	32
**8**	–	–	–	**2+4+5**	–	64	64
**9**	–	256	256	**2+4+5+6**	–	64	64
Amikacin	1	1	1	**1+2+3+4 +5+6+7**	256	64	32

**Notes.**

“–” indicate inactive (MIC > 256 µg/mL).

### Antioxidant assay

Compounds **1**–**9** were also subjected to screening for possible antioxidant activity by the DPPH free radical scavenging assay (Table 4). As a result, compound **6** showed the best antioxidant activity with an IC_50_ value of 17.62 µg/mL. Compounds **5** and **2** also displayed stronger antioxidant activity than BHT with the IC_50_ value of 25.93 µg/mL and 39.03 µg/mL, respectively. Compound **4** displayed relatively weak antioxidant activity with an IC_50_ value of 130.68 µg/mL. Other compounds indicated inactivity and the IC_50_ values were larger than 200 µg/mL. Combinations of **5** + **6**, **2** + **4** + **5** and **2** + **4** + **5** + **6** displayed synergistic effect and the antioxidant activities were better than the single compound. While combinations of **2** + **1**, **2** + **3** and **2** + **4** showed minus effect and the IC_50_ values were all larger than 200 µg/mL.

**Table 4:** Antioxidant activities.

**Compound**	**IC_50_(µg/mL)**	**Compound**	**IC_50_(µg/mL)**
**1**	–	**2 + 1**	–
**2**	39.03 ± 0.64	**2 + 3**	–
**3**	–	**2 + 4**	–
**4**	130.68 ± 9.28	**2 + 5**	28.40 ± 0.16
**5**	25.93 ± 1.06	**2 + 6**	23.33 ± 0.77
**6**	17.62 ± 0.61	**2 + 7**	176.56 ± 3.62
**7**	–	**5 + 6**	3.37 ± 0.11
**8**	–	**2+4+5**	16.45 ± 0.04
**9**	–	**2+4+5+6**	13.59 ± 0.08
BHT	40.93 ± 0.89	**1+2+3+4+5+6+7**	32.60 ± 0.04

**Notes.**

“–” indicates inactive (IC_50_ > 200 µg/mL).

## Discussion

In our previous study, the EtOAc extract of *Aspergillus candidus* Bdf-2 from *Blaptica dubia* displayed the best antibacterial activity and excellent antioxidant activity among the isolated strains (Shan et al., 2019). Thus *Aspergillus candidus* Bdf-2 was selected for the further isolation and identification of the secondary metabolites. Based on the results presented here, we describe a new *p*-terphenyl derivative 4″-deoxy- 2′-methoxyterphenyllin (**1**), along with eight known compounds were isolated from the insect-derived strain of the fungus *Aspergillus candidus* Bdf-2. Compounds **1**–**6** were all *p*-terphenyl derivatives and compound 7 was candidusin derivatives. Candidusin can be deduced to be a cyclization product of terphenyllin between C-6 and C-2′ via an oxygen atom (Wang et al., 2017). Based on the antibacterial and antioxidant activities results (Tables 3 and 4), some structure–activity relationships among the *p*-terphenyl derivatives were found. Compound **6** displayed the strongest antibacterial and antioxidant activities and there were five hydroxyls in its structure. Compound **5** also showed good antibacterial and antioxidant activities and had four hydroxyls. Compounds **1** and **3** only had one hydroxyl in their structures and didn’t show any bioactivity at the test concentrations. Above all, the hydroxyl was presumed as the active functional group in *p*-terphenyl derivatives and the strength of activity depending on the number of hydroxyl groups. In addition, compound **9** showed no antibacterial or antioxidant activity, which was consistent with the results reported in the literature (Zin et al., 2016; Kumla et al., 2017).

Compound **2** which was the dominant compound didn’t show strong individual bioactivity in this study. To date, there have been a lot of reports about the biological activities of single *p*-terphenyl derivatives, while no reports on the synergistic antibacterial and antioxidant activities. Hence, the synergistic effects of compound **2** with other compounds were also investigated. According to the results in Tables 3 and 4, parts of *p*-terphenyl derivatives displayed obvious synergistic effects. In this study, the compounds in different combinations were just mixed together at the same concentration and volume. Different proportions of the compounds may have a great impact on bioactivity. Therefore, the relative content of different compounds in metabolites of *Aspergillus candidus* Bdf-2 would deserve further study.

## Conclusions

*Aspergillus candidus* Bdf-2 associated with *Blaptica dubia* was a strain rich in *p*-terphenyl derivatives and terphenyllin (**2**) was the dominant compound in the EtOAc crude extracts. Antibacterial and antioxidant activities of single compound were not as good as synergistic effect. Maybe this was a good explanation for why the crude extract had good bioactivities. We can still find some patterns in the antibacterial and antioxidant activities of monomer compounds. The hydroxyl was presumed as the active functional group in *p*-terphenyl derivatives and the strength of activity depending on the number of hydroxyl groups. Compound **6** displayed the best antibacterial and antioxidant activities, which may be a promising candidate for further studies in the development of a potent antibacterial and antioxidant.

##  Supplemental Information

10.7717/peerj.8221/supp-1 File S1 Supplemental file Figure S1 Colony of *Aspergillus candidus* Bdf-2.
Figure S2–S5 Phylogenetic tree of *Aspergillus candidus* Bdf-2 based on four different gene sequence
Figure S6–S38 Raw data for the UV, IR, HR-ESI-MS and NMR of compounds 1–9.

10.7717/peerj.8221/supp-2 Supplemental Information 1 Raw data of Table 3 MIC values of the compounds and positive control were obtained from the sterile 96-well plates directly, so we didn’t provided the statistics.

10.7717/peerj.8221/supp-3 Supplemental Information 2 Raw data for antioxidant activities (IC50) The DPPH clearance rate of compounds 1,3,7,8,9, 2+1, 2+3 and 2+4 were less than 50% at the highest test concentration, so their IC50 values were not calculated.

10.7717/peerj.8221/supp-4 Supplemental Information 3 Sequences uploaded to GenBank

## Additional Information and Declarations

### DNA Deposition

The following information was supplied regarding the deposition of DNA sequences: The isolate was identified as *Aspergillus candidus* by analysis of its morphological characteristics and the rDNA gene internal transcribed spacer (ITS) sequence (GenBank: MH681592), as well as beta-tubulin (tub2) gene sequence (GenBank: MN533958), RNA polymerase II second largest subunit (RPB2) gene sequence (GenBank: MN533959) and large subunit ribosomal RNA gene sequence (GenBank: MN533960).

### Data Availability

The following information was supplied regarding data availability: The raw measurements are available in the Supplemental Files. The Bdf2 strain is available at the China Center for Type Culture Collection: CCTCC AF 2019013. http://www.cctcc.org/sci/microbe_common/search_result.php?ptzyh=1542C0001AF2019013.
