# Supplementary material for: A new p-terphenyl derivative from the insect-derived fungus Aspergillus candidus Bdf-2 and the synergistic effects of terphenyllin
Source: PeerJ. 2020 Jan 2;8:e8221. doi: 10.7717/peerj.8221 (PMC6942676; doi:10.7717/peerj.8221)
Supplement: File S1 — Figure S1 Colony of Aspergillus candidus Bdf-2. Figure S2–S5 Phylogenetic tree of Aspergillus candidus Bdf-2 based on four different gene sequence Figure S6–S38 Raw data for the UV, IR, HR-ESI-MS and NMR of compounds 1–9. [file peerj-08-8221-s001.docx]

Supporting Information

**A new *p*-terphenyl derivative from the insect-derived fungus *Aspergillus* sp. Bdf-2 and the synergistic effects of terphenyllin**

Tijiang Shan^1†^, Yuyang Wang^1†^, Song Wang^1^, Yunying Xie^2^, Zehua Cui^3^, Chunyin Wu^1^, Jian Sun^3^, Jun Wang^1^ and Ziling Mao^1*^

^1^ Guangdong Key Laboratory for Innovative Development and Utilization of Forest Plant Germplasm, College of Forestry and Landscape Architecture, South China Agricultural University, Guangzhou,Guangdong, China

^2^ Institute of Medicinal Biotechnology, Chinese Academy of Medical Sciences & Peaking Union Medical College, Beijing, China

^3^ National Risk Assessment Laboratory for Antimicrobial Resistance of Animal Original Bacteria, South China Agricultural University, Guangzhou, Guangdong, China

Corresponding Author:

Ziling Mao^1^

483 Wushan Road, Tianhe District, Guangzhou,Guangdong, 510642, China

Email address: zlmao@scau.edu.cn

^†^These authors contributed equally to this work.

**Contents**

[Figure S1 Colony of *Aspergillus candidus* Bdf-2 4](#_Toc22241139)

[Figure S2 Phylogenetic tree of *Aspergillus candidus* Bdf-2 based on the rDNA-ITS sequence 4](#_Toc22241140)

[Figure S3 Phylogenetic tree of *Aspergillus candidus* Bdf-2 based on the beta-tubulin (tub2) gene sequence 5](#_Toc22241141)

[Figure S4 Phylogenetic tree of *Aspergillus candidus* Bdf-2 based on the RNA polymerase II second largest subunit (RPB2) gene sequence 5](#_Toc22241142)

[Figure S5 Phylogenetic tree of *Aspergillus candidus* Bdf-2 based on the large subunit ribosomal RNA gene sequence 6](#_Toc22241143)

[Figure S6 UV spectrum of 4''-Dehydroxy-2'-methoxyterphenyllin (1) 6](#_Toc22241144)

[Figure S7 IR spectrum of 4''-Dehydroxy-2'-methoxyterphenyllin (1) 7](#_Toc22241145)

[Figure S8 HR-ESI-MS spectrum of 4''-Dehydroxy-2'-methoxyterphenyllin (1) 8](#_Toc22241146)

[Figure S9 ^1^H NMR spectrum of 4''-Dehydroxy-2'-methoxyterphenyllin (1) (Acetone-*d*_6_, 600 MHz) 8](#_Toc22241147)

[Figure S10 ^13^C NMR spectrum of 4''-Dehydroxy-2'-methoxyterphenyllin (1) (Acetone-*d*_6_, 151 MHz) 9](#_Toc22241148)

[Figure S11 HSQC spectrum of 4''-Dehydroxy-2'-methoxyterphenyllin (1) (Acetone-*d*_6_) 9](#_Toc22241149)

[Figure S12 HMBC spectrum of 4''-Dehydroxy-2'-methoxyterphenyllin (1) (Acetone-*d*_6_) 10](#_Toc22241150)

[Figure S13 ^1^H-^1^H COSY spectrum of 4''-Dehydroxy-2'-methoxyterphenyllin (1) (Acetone-*d*_6_) 10](#_Toc22241151)

[Figure S14 NOESY spectrum of 4''-Ddeoxy-2'-methoxyterphenyllin (1) (Acetone-*d*_6_) 11](#_Toc22241152)

[Figure S15 HR-ESI-MS spectrum of Terphenyllin (2) 11](#_Toc22241153)

[Figure S16 ^1^H NMR spectrum of Terphenyllin (2) (DMSO-*d*_6_, 600 MHz) 12](#_Toc22241154)

[Figure S17 ^13^C NMR spectrum of Terphenyllin (2) (DMSO-*d*_6_, 151 MHz) 12](#_Toc22241155)

[Figure S18 HR-ESI-MS spectrum of 4, 4''-Deoxyterphenyllin (3) 13](#_Toc22241156)

[Figure S19 ^1^H NMR spectrum of 4, 4''-Deoxyterphenyllin (3) (CDCl_3_, 600 MHz) 13](#_Toc22241157)

[Figure S20 ^13^C NMR spectrum of 4, 4''-Deoxyterphenyllin (3) (CDCl_3_, 151 MHz) 14](#_Toc22241158)

[Figure S21 HR-ESI-MS spectrum of 4″-Ddeoxyterphenyllin (4) 14](#_Toc22241159)

[Figure S22 ^1^H NMR spectrum of 4″-Ddeoxyterphenyllin (4) (Acetone-*d*_6_, 600 MHz) 15](#_Toc22241160)

[Figure S23 ^13^C NMR spectrum of 4″-Ddeoxyterphenyllin (4) (Acetone-*d*_6_, 151 MHz) 15](#_Toc22241161)

[Figure S24 HR-ESI-MS spectrum of 3''-Hydroxyterphenyllin (5) 16](#_Toc22241162)

[Figure S25 ^1^H NMR spectrum of 3''-Hydroxyterphenyllin (5) (CD_3_OD, 600 MHz) 16](#_Toc22241163)

[Figure S26 ^13^C NMR spectrum of 3''-Hydroxyterphenyllin (5) (CD_3_OD, 151 MHz) 17](#_Toc22241164)

[Figure S27 HR-ESI-MS spectrum of 3, 3''-Dihydroxyterphenyllin (6) 17](#_Toc22241165)

[Figure S28 ^1^H NMR spectrum of 3, 3''- Dihydroxyterphenyllin (6) (CD_3_OD, 600 MHz) 18](#_Toc22241166)

[Figure S29 ^13^C NMR spectrum of 3, 3''- Dihydroxyterphenyllin (6) (CD_3_OD, 151 MHz) 18](#_Toc22241167)

[Figure S30 HR-ESI-MS spectrum of Candidusin A (7) 19](#_Toc22241168)

[Figure S31 ^1^H NMR spectrum of Candidusin A (7) (CD_3_OD, 600 MHz) 19](#_Toc22241169)

[Figure S32 ^13^C NMR spectrum of Candidusin A (7) (CD_3_OD, 151 MHz) 20](#_Toc22241170)

[Figure S33 HR-ESI-MS spectrum of Dechlorochlorflavonin (8) 20](#_Toc22241171)

[Figure S34 ^1^H NMR spectrum of Dechlorochlorflavonin (8) (CDCl_3_, 600 MHz) 21](#_Toc22241172)

[Figure S35 ^13^C NMR spectrum of Dechlorochlorflavonin (8) (CDCl_3_, 151 MHz) 21](#_Toc22241173)

[Figure S36 HR-ESI-MS spectrum of Fellutanine A (9) 22](#_Toc22241174)

[Figure S37 ^1^H NMR spectrum of Fellutanine A (9) (CD_3_OD, 600 MHz) 22](#_Toc22241175)

[Figure S38 ^13^C NMR spectrum of Fellutanine A (9) (CD_3_OD, 151 MHz) 23](#_Toc22241176)


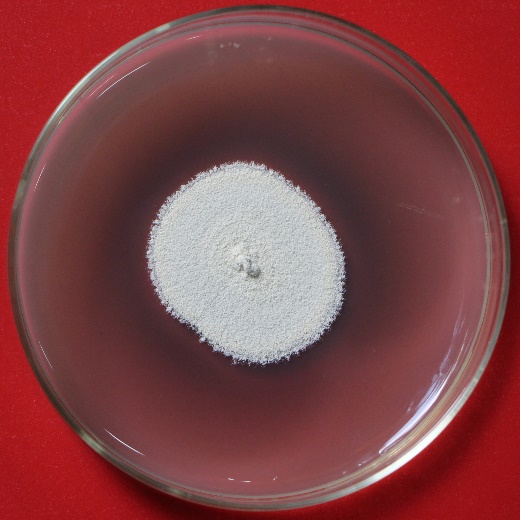


# Figure S1 Colony of *Aspergillus candidus* Bdf-2


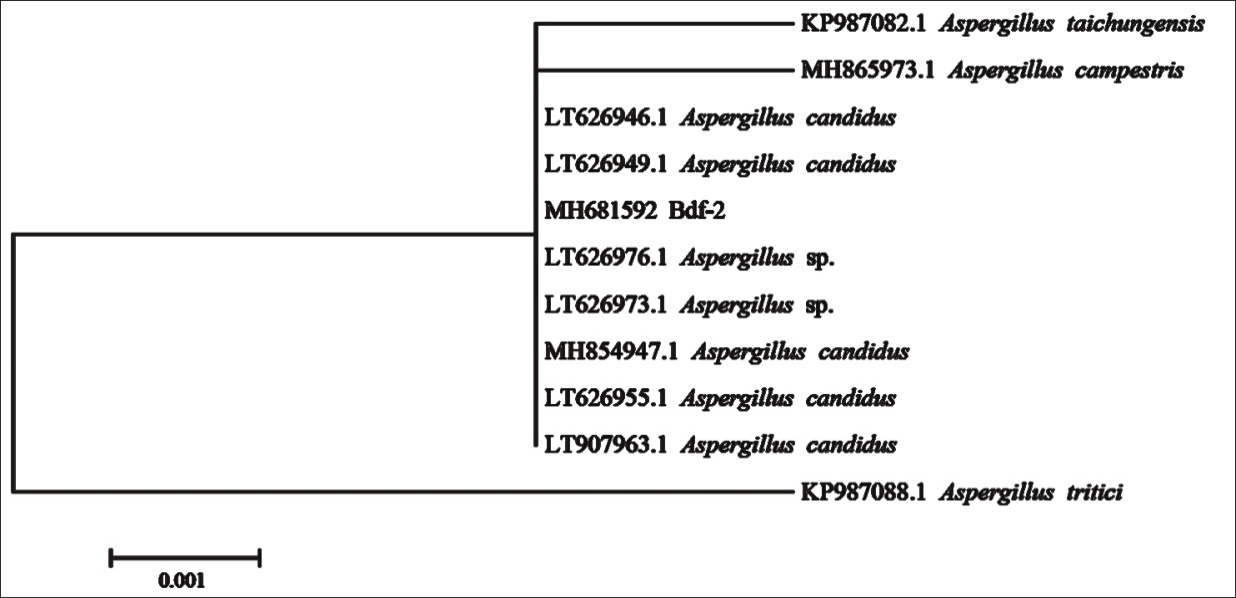


# Figure S2 Phylogenetic tree of *Aspergillus candidus* Bdf-2 based on the rDNA-ITS sequence


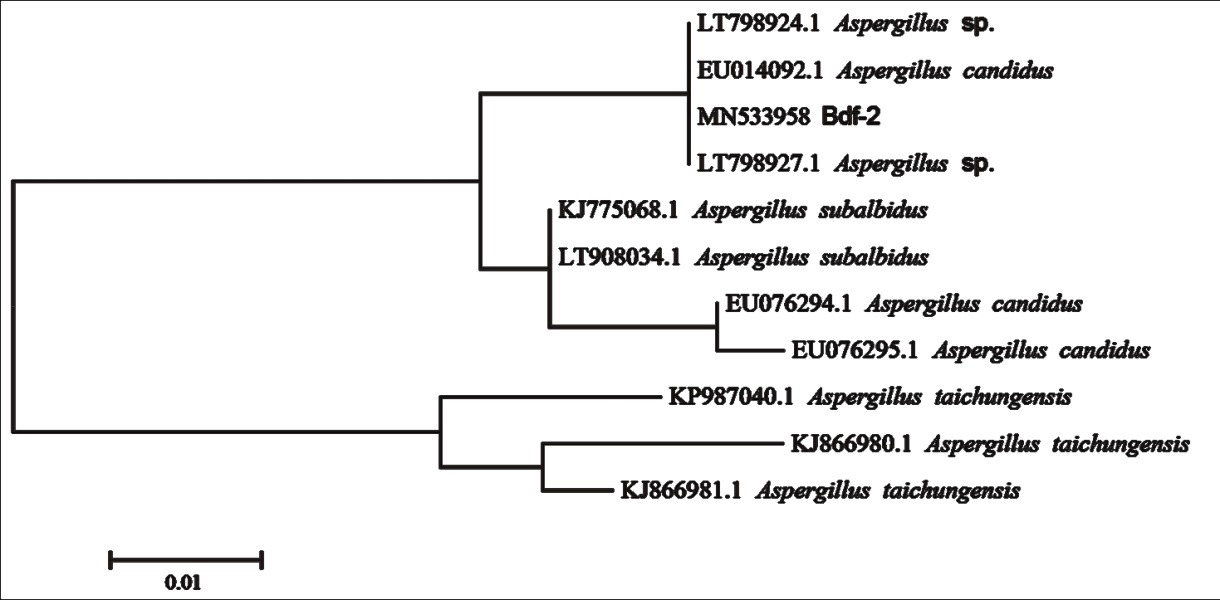


# Figure S3 Phylogenetic tree of *Aspergillus candidus* Bdf-2 based on the beta-tubulin (tub2) gene sequence


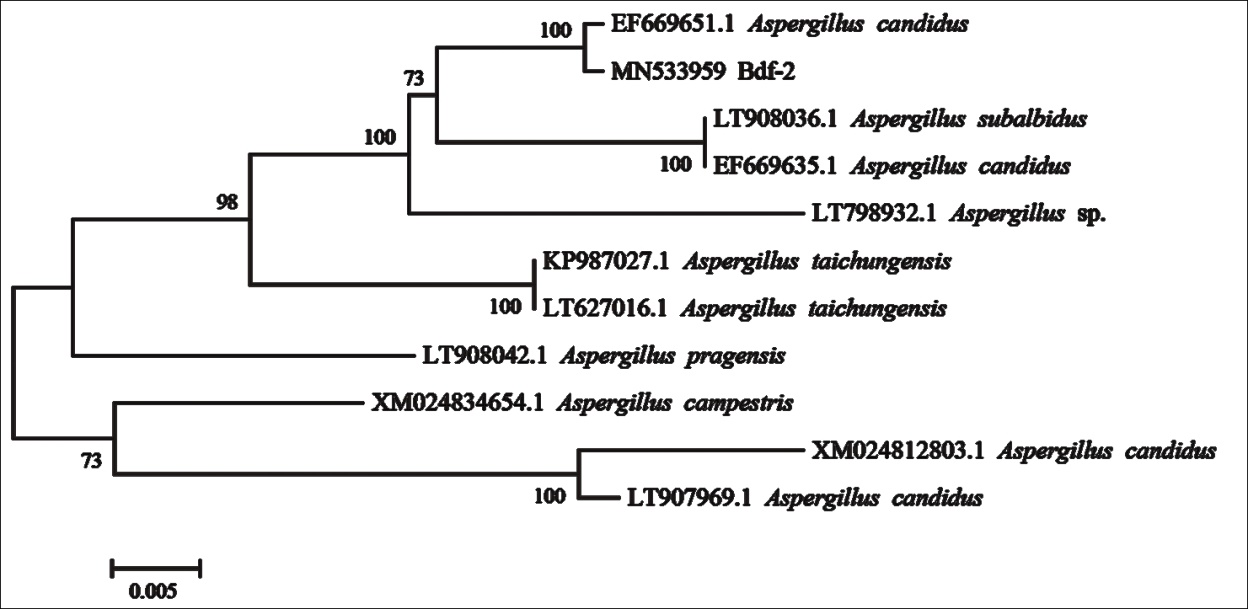


# Figure S4 Phylogenetic tree of *Aspergillus candidus* Bdf-2 based on the RNA polymerase II second largest subunit (RPB2) gene sequence


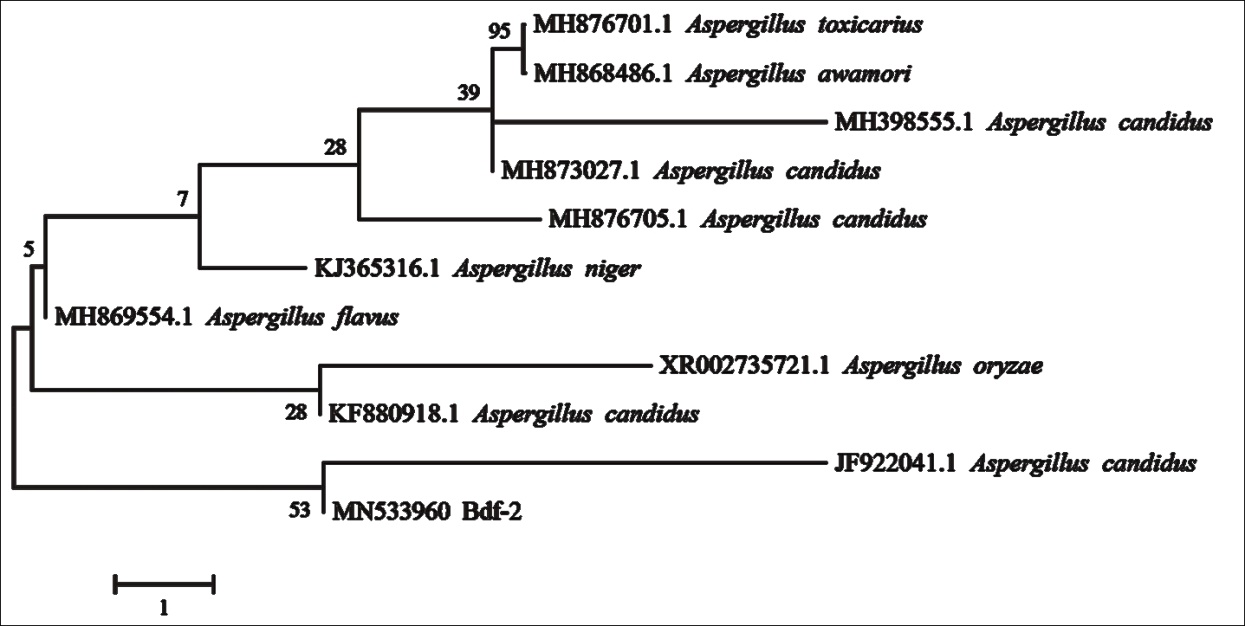


# Figure S5 Phylogenetic tree of *Aspergillus candidus* Bdf-2 based on the large subunit ribosomal RNA gene sequence


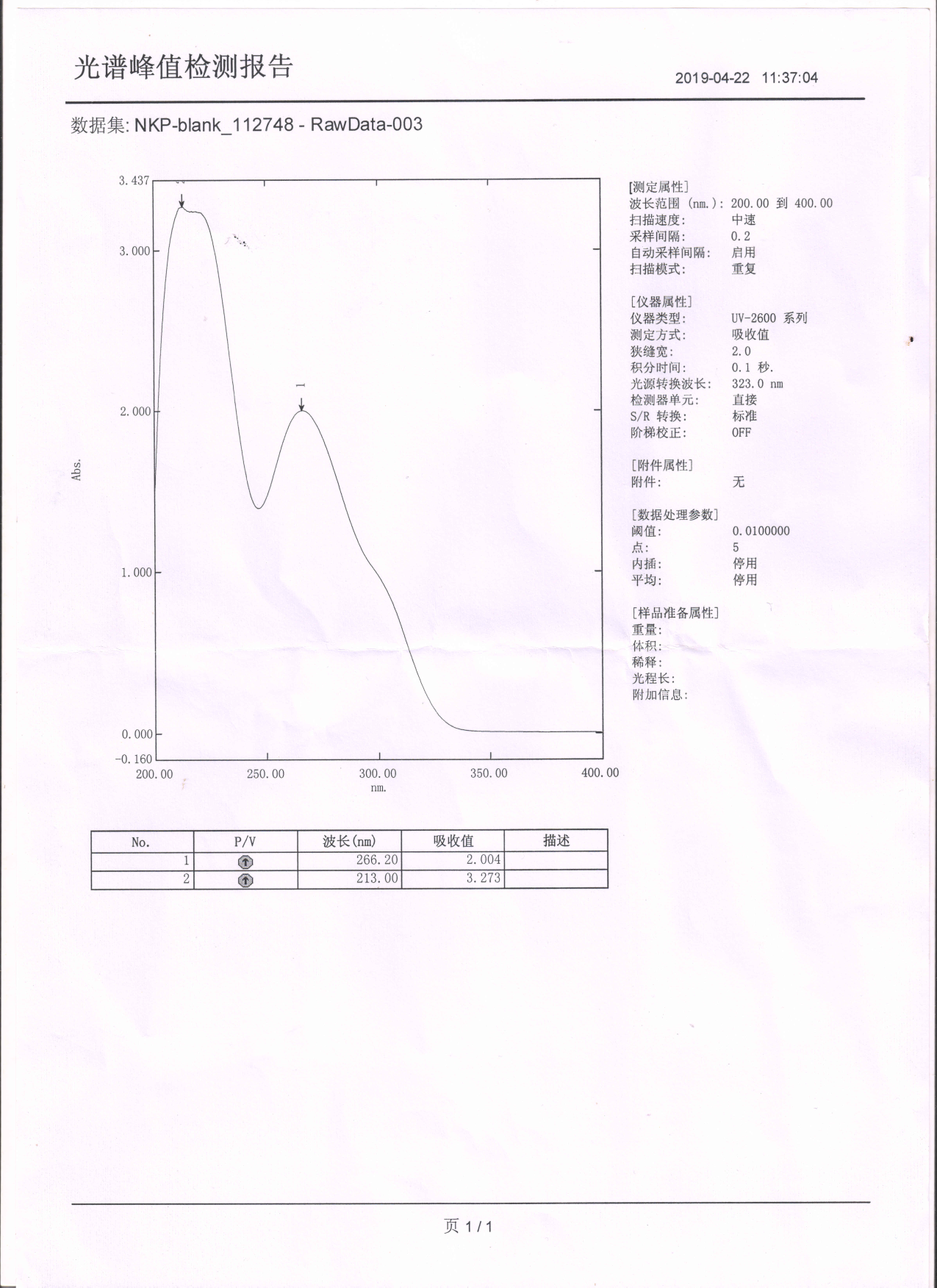


# Figure S6 UV spectrum of 4''-Dehydroxy-2'-methoxyterphenyllin (1)


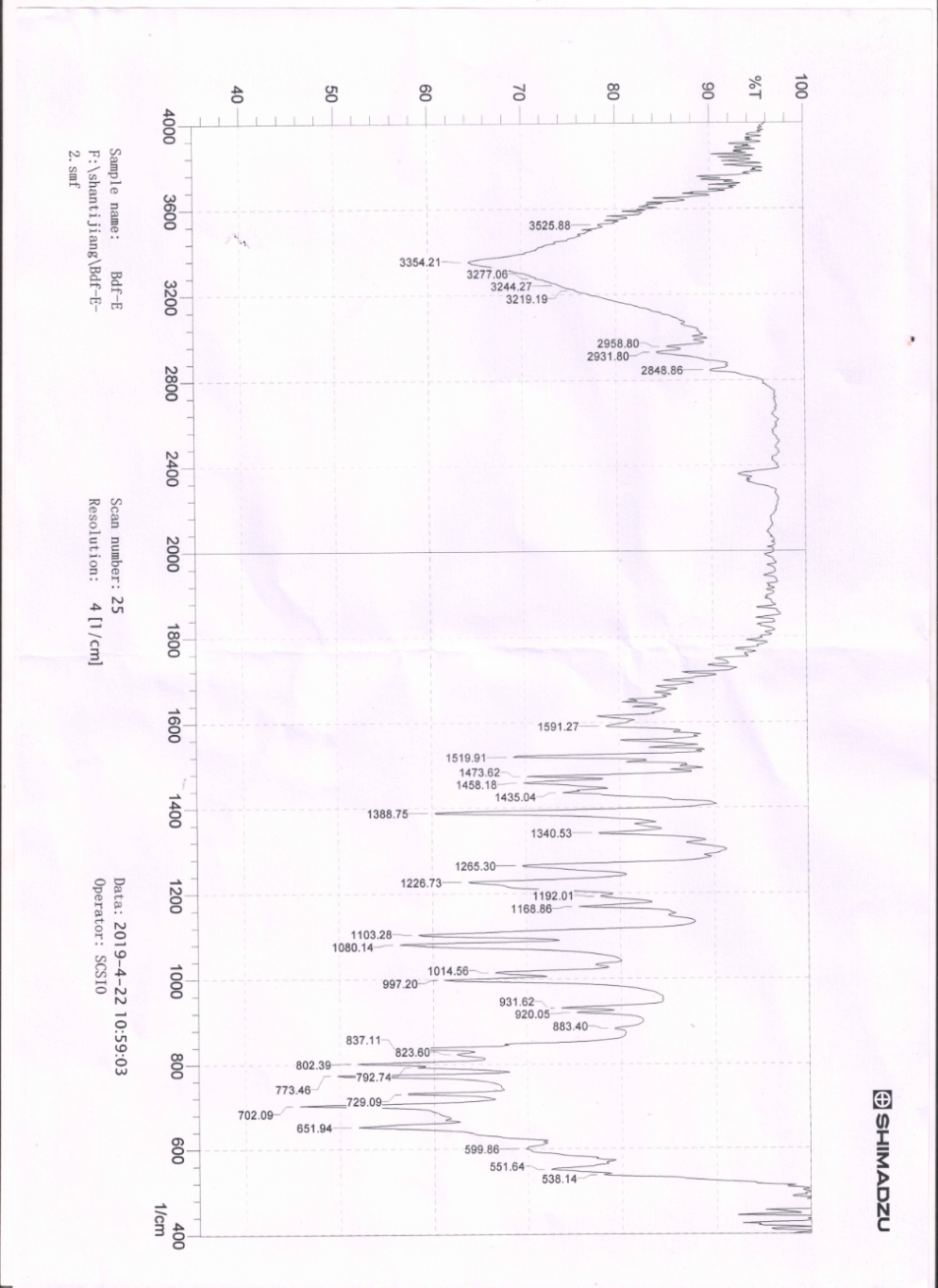


# Figure S7 IR spectrum of 4''-Dehydroxy-2'-methoxyterphenyllin (1)


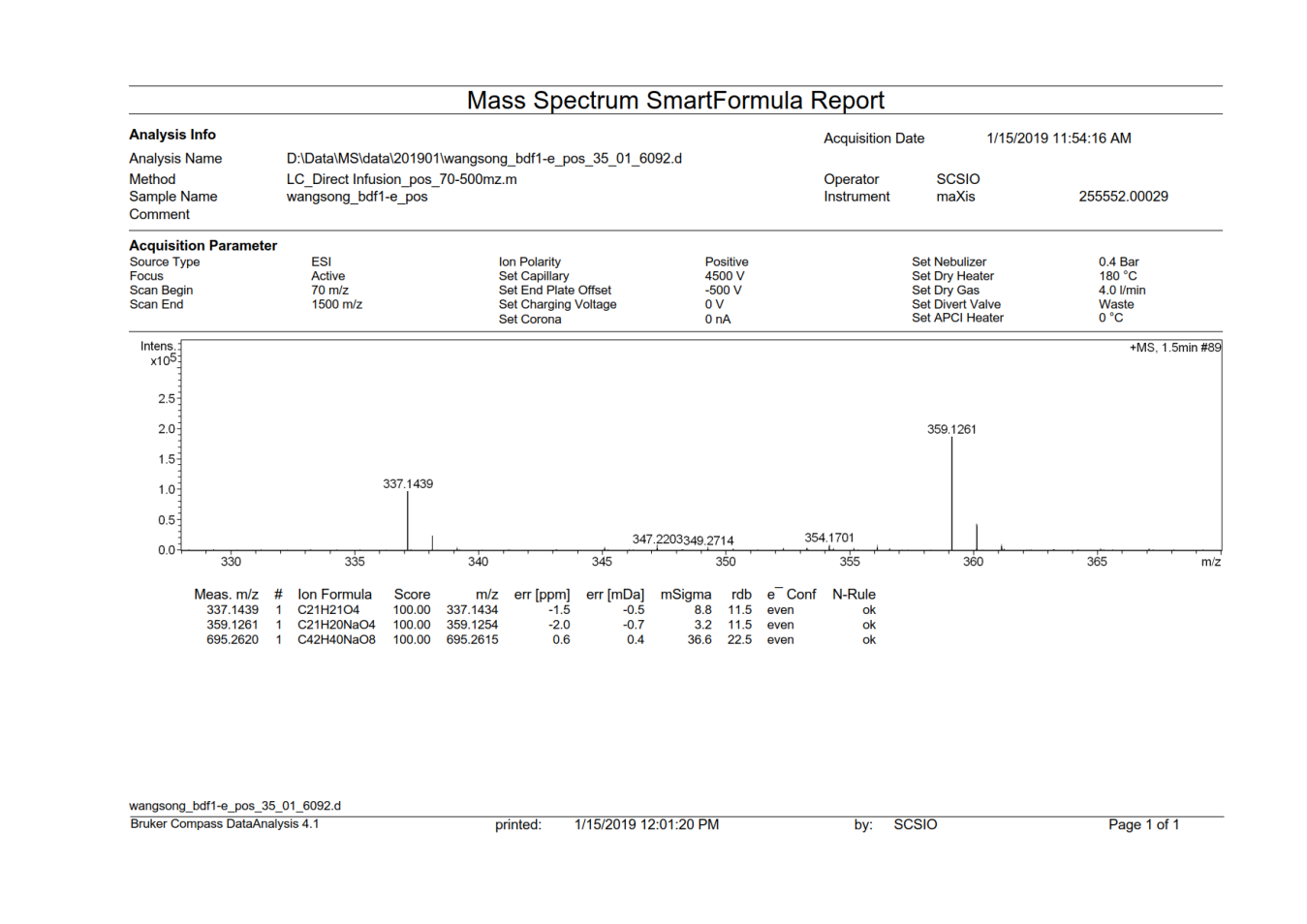


# Figure S8 HR-ESI-MS spectrum of 4''-Dehydroxy-2'-methoxyterphenyllin (1)


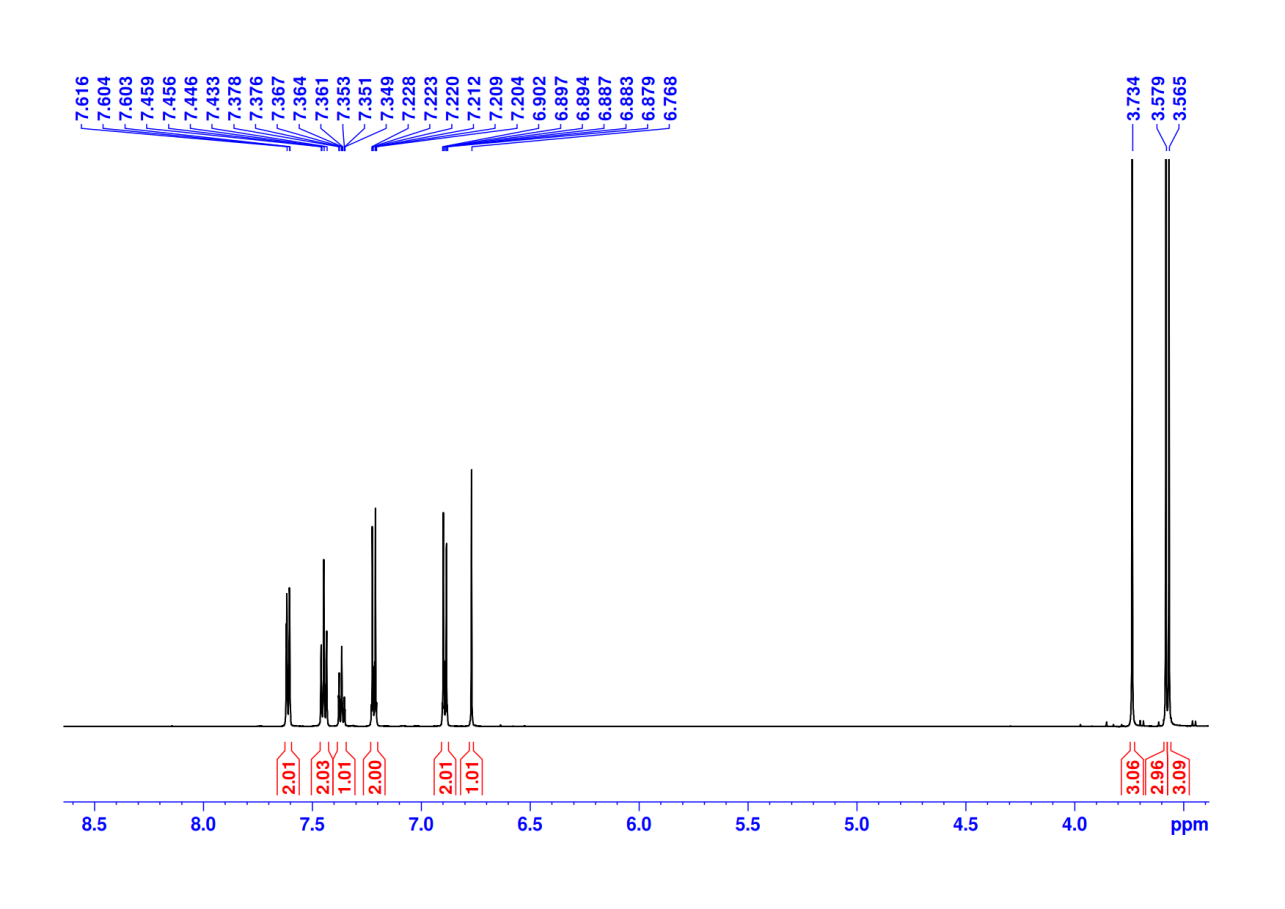


# Figure S9 ^1^H NMR spectrum of 4''-Dehydroxy-2'-methoxyterphenyllin (1) (Acetone-*d*_6_, 600 MHz)


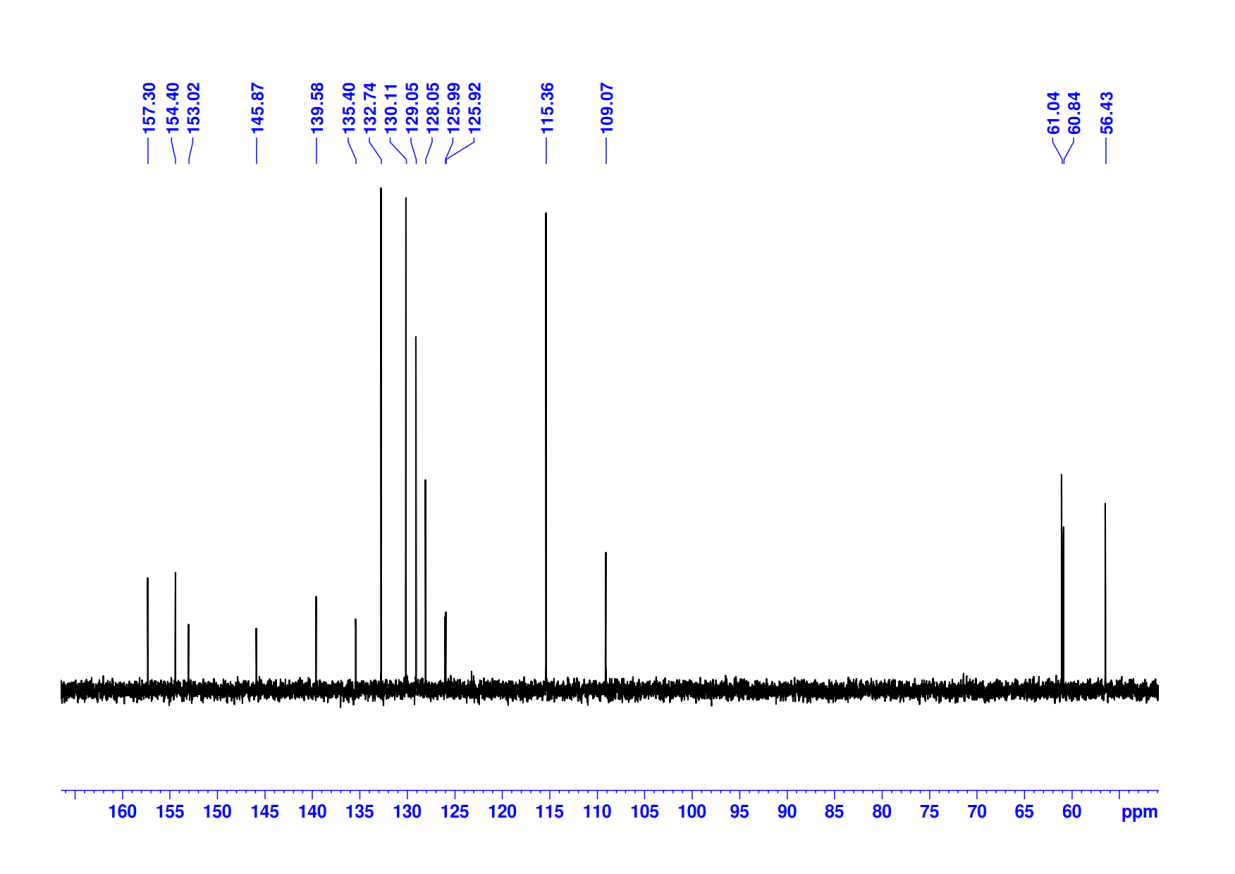


# Figure S10 ^13^C NMR spectrum of 4''-Dehydroxy-2'-methoxyterphenyllin (1) (Acetone-*d*_6_, 151 MHz)


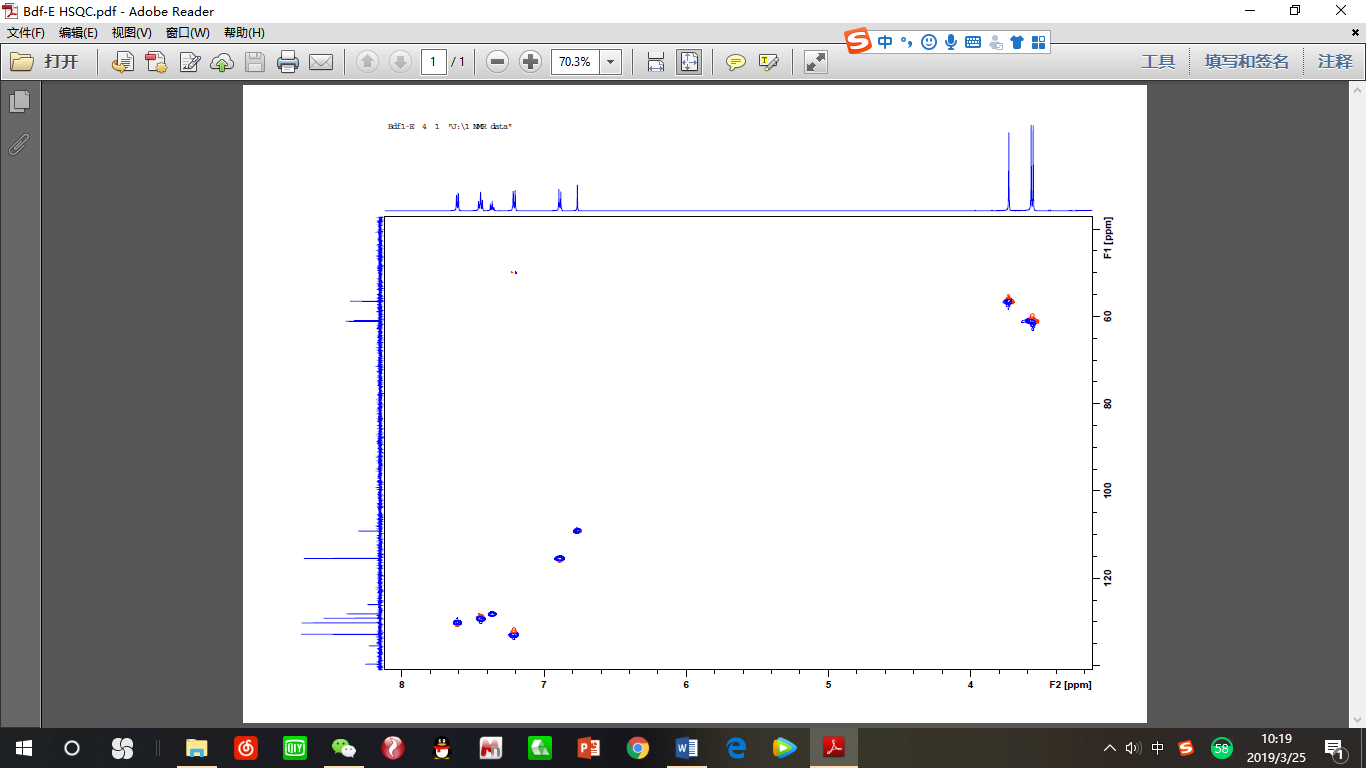


# Figure S11 HSQC spectrum of 4''-Dehydroxy-2'-methoxyterphenyllin (1) (Acetone-*d*_6_)


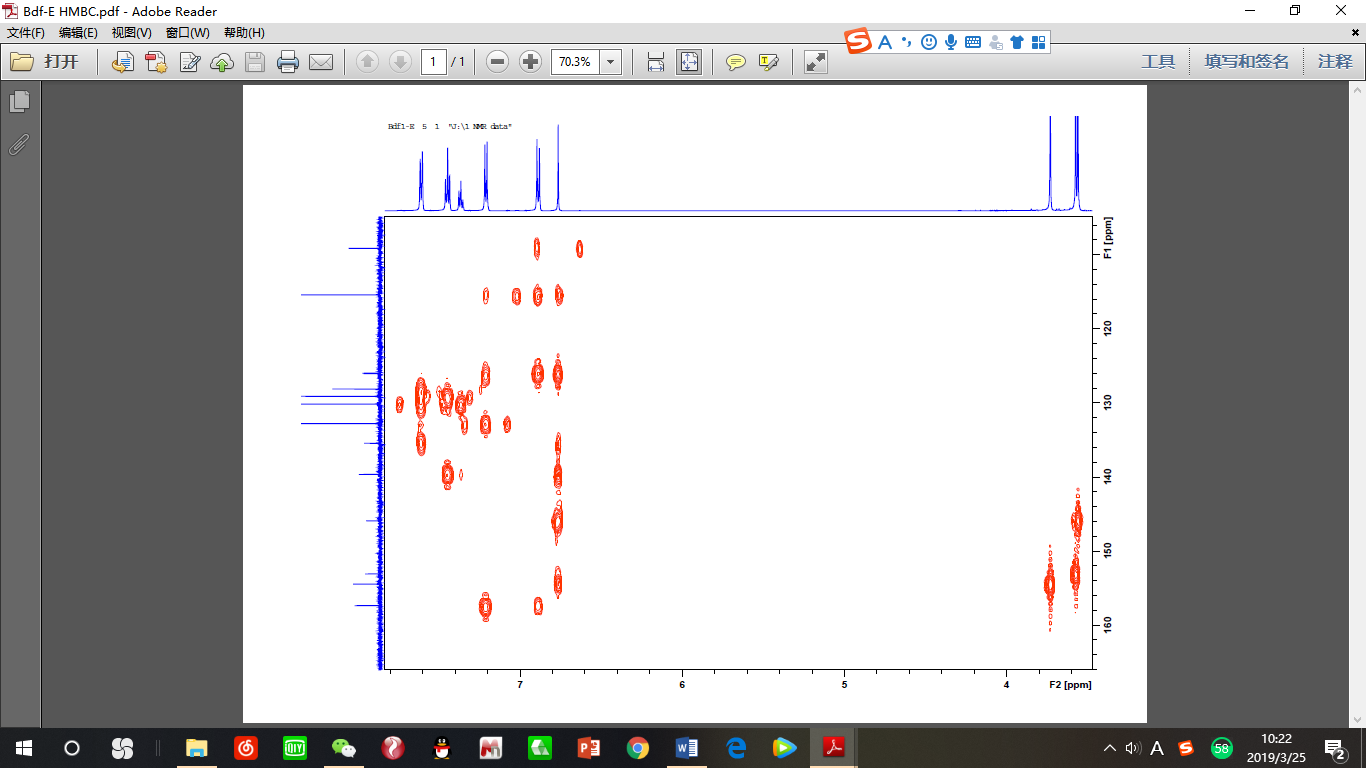


# Figure S12 HMBC spectrum of 4''-Dehydroxy-2'-methoxyterphenyllin (1) (Acetone-*d*_6_)


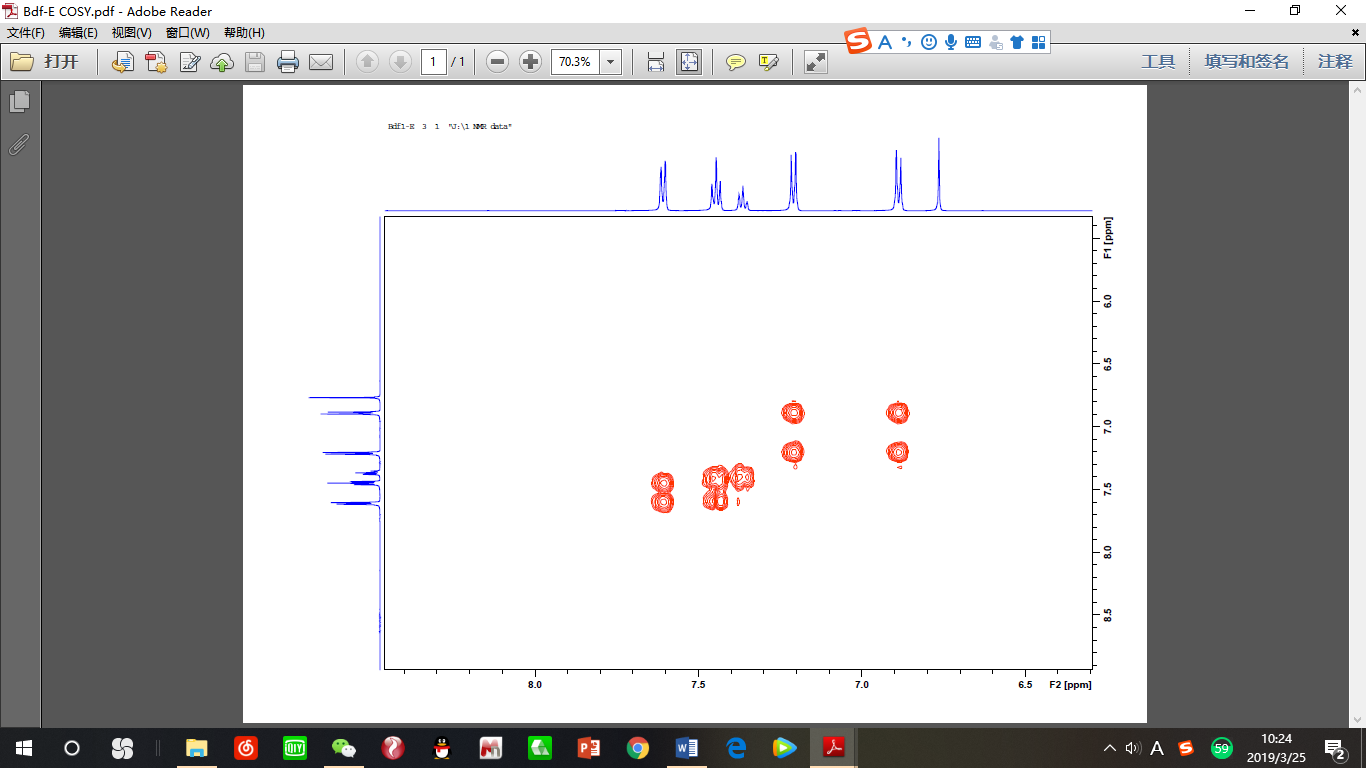


# Figure S13 ^1^H-^1^H COSY spectrum of 4''-Dehydroxy-2'-methoxyterphenyllin (1) (Acetone-*d*_6_)


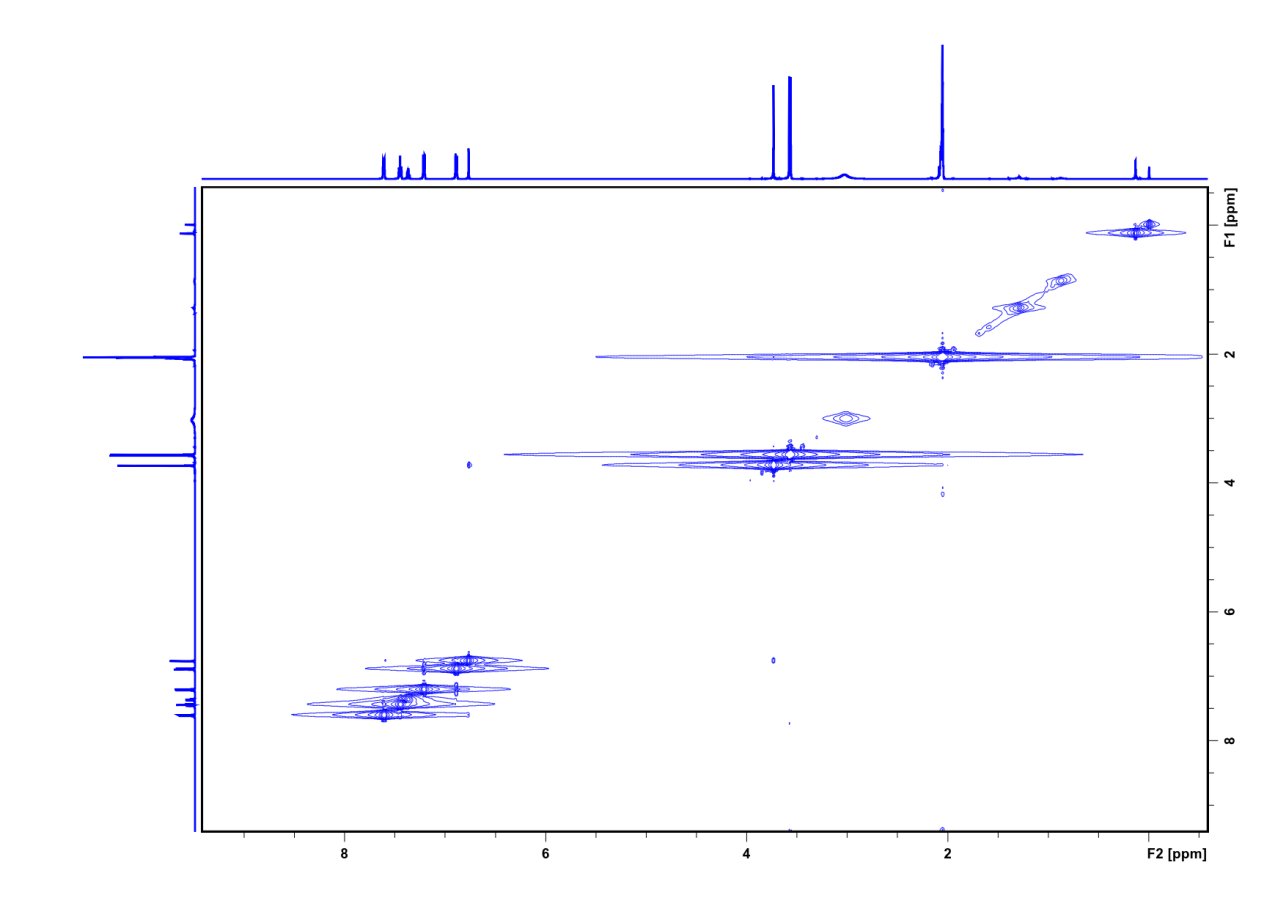


# Figure S14 NOESY spectrum of 4''-Ddeoxy-2'-methoxyterphenyllin (1) (Acetone-*d*_6_)


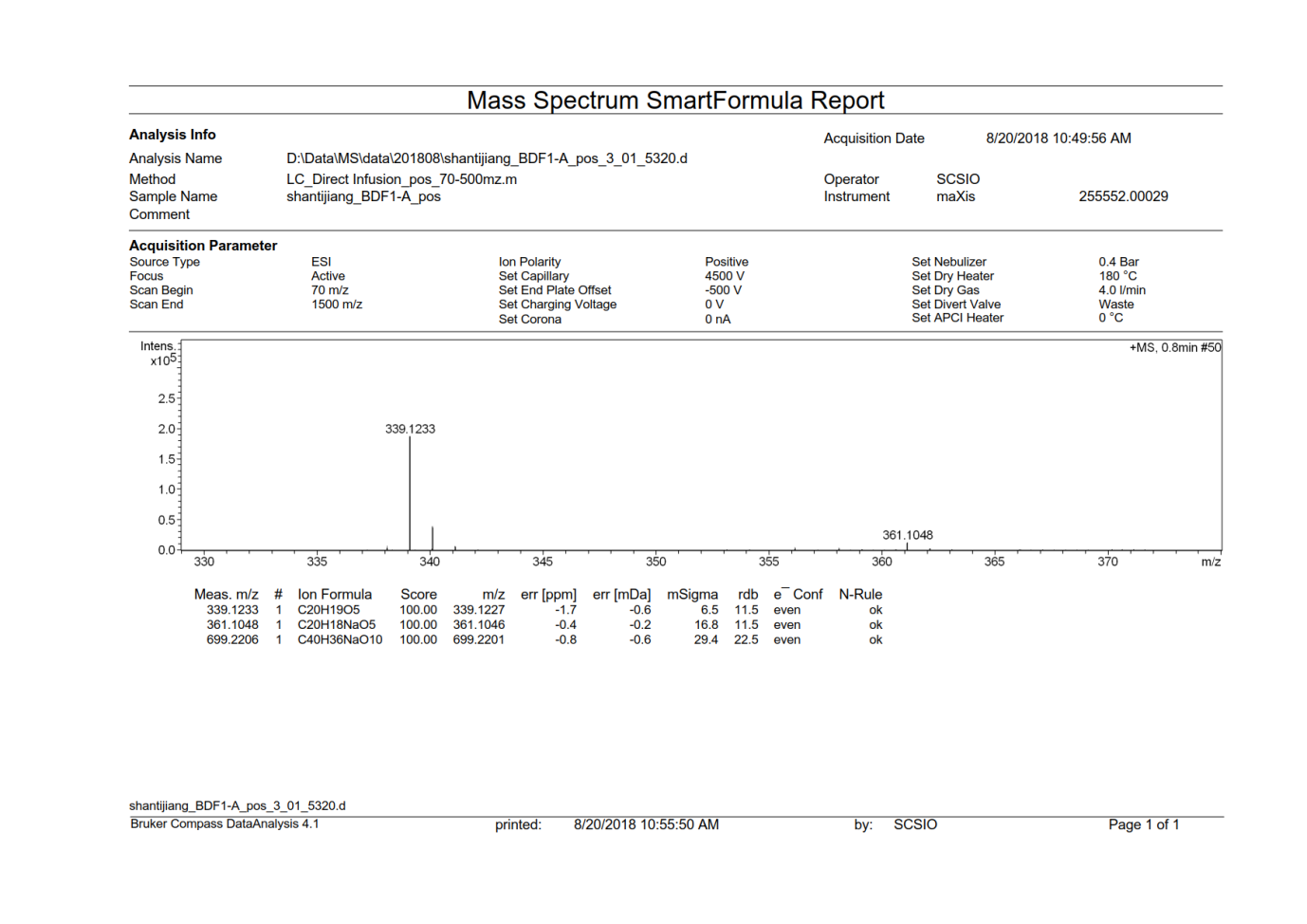


# Figure S15 HR-ESI-MS spectrum of Terphenyllin (2)


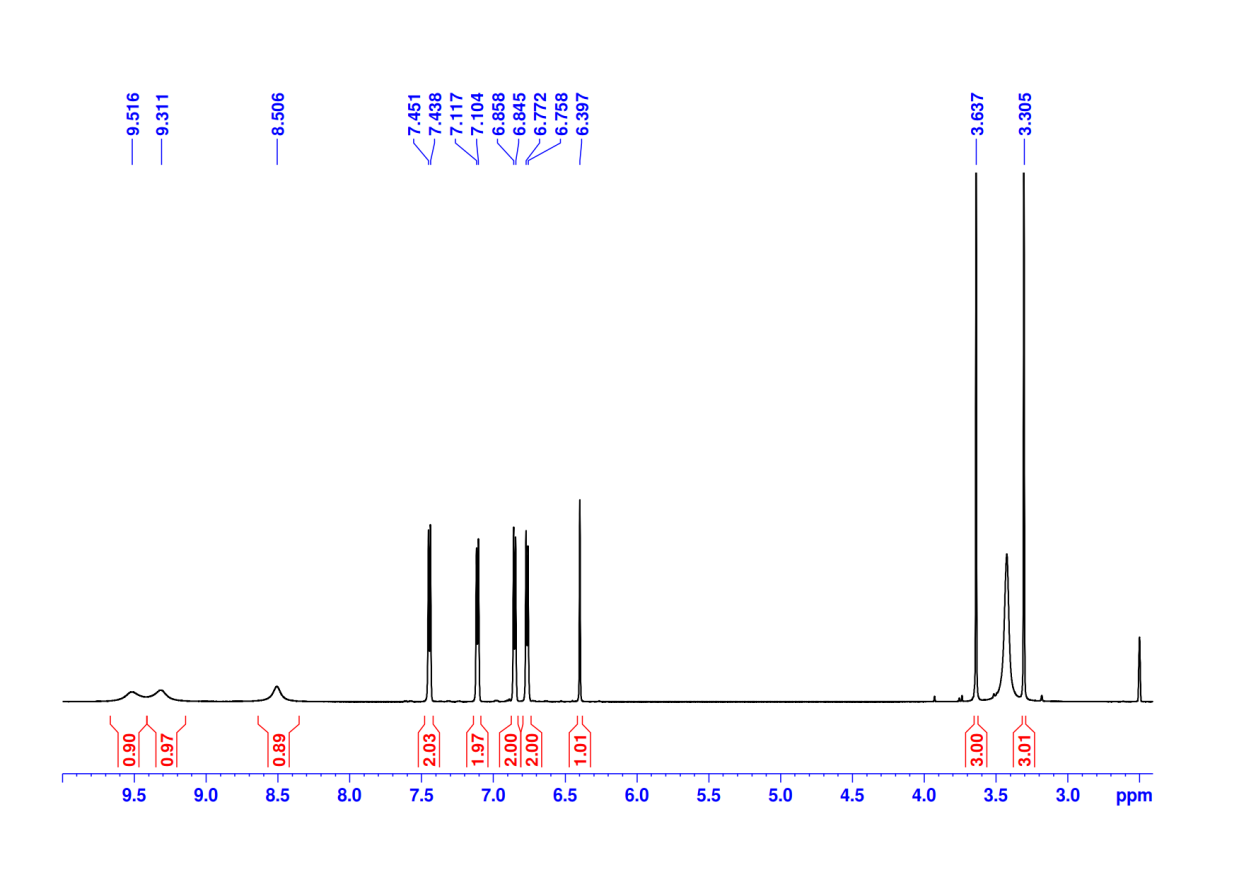


# Figure S16 ^1^H NMR spectrum of Terphenyllin (2) (DMSO-*d*_6_, 600 MHz)


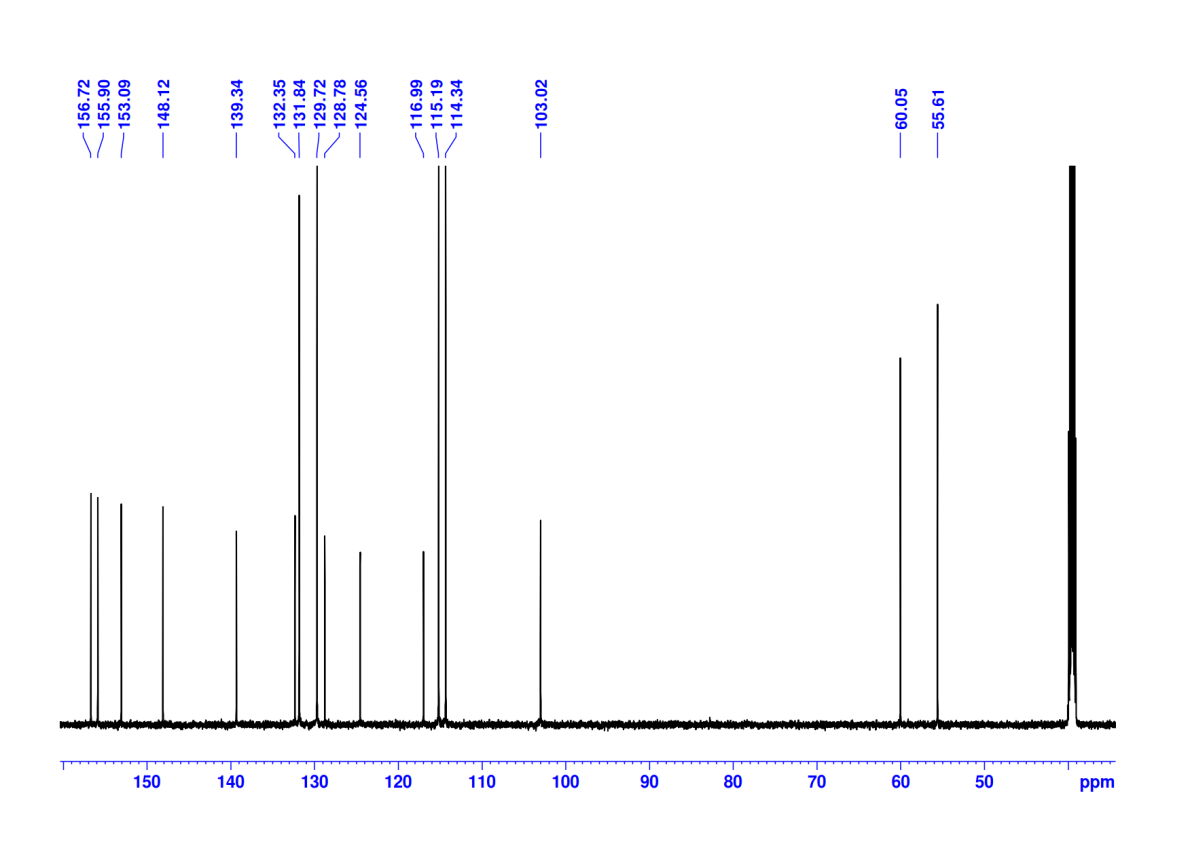


# Figure S17 ^13^C NMR spectrum of Terphenyllin (2) (DMSO-*d*_6_, 151 MHz)


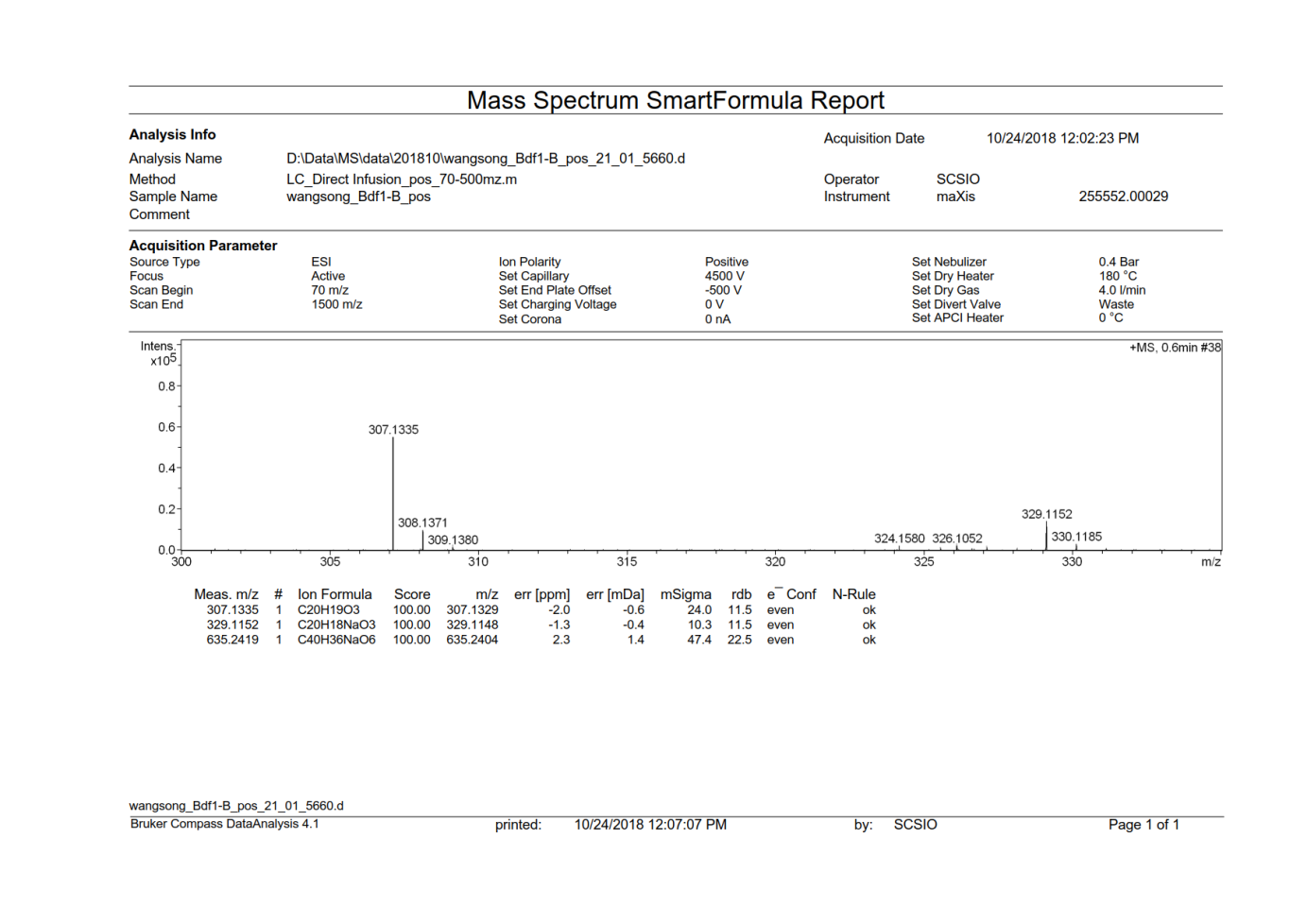


# Figure S18 HR-ESI-MS spectrum of 4, 4''-Deoxyterphenyllin (3)


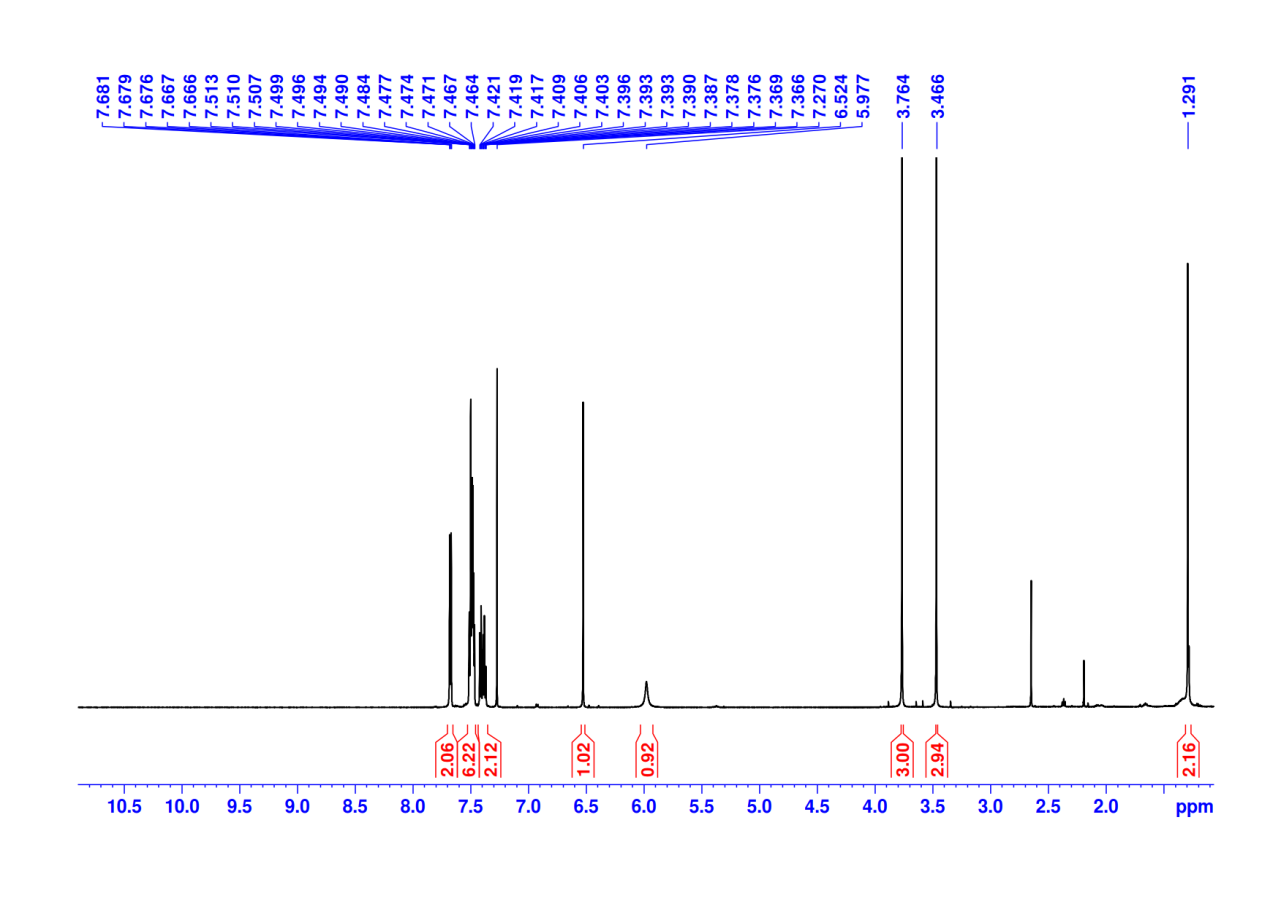


# Figure S19 ^1^H NMR spectrum of 4, 4''-Deoxyterphenyllin (3) (CDCl_3_, 600 MHz)

**
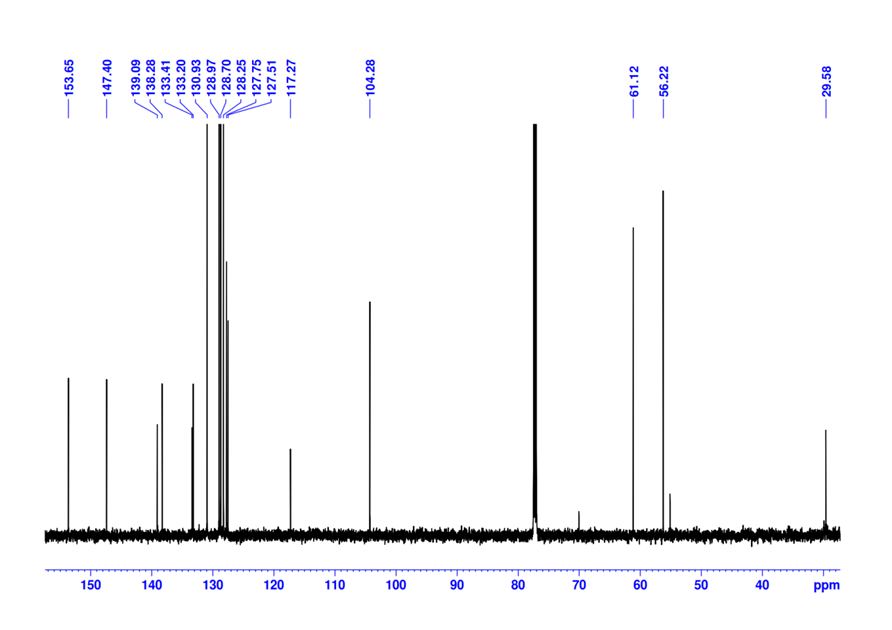
**

# Figure S20 ^13^C NMR spectrum of 4, 4''-Deoxyterphenyllin (3) (CDCl_3_, 151 MHz)


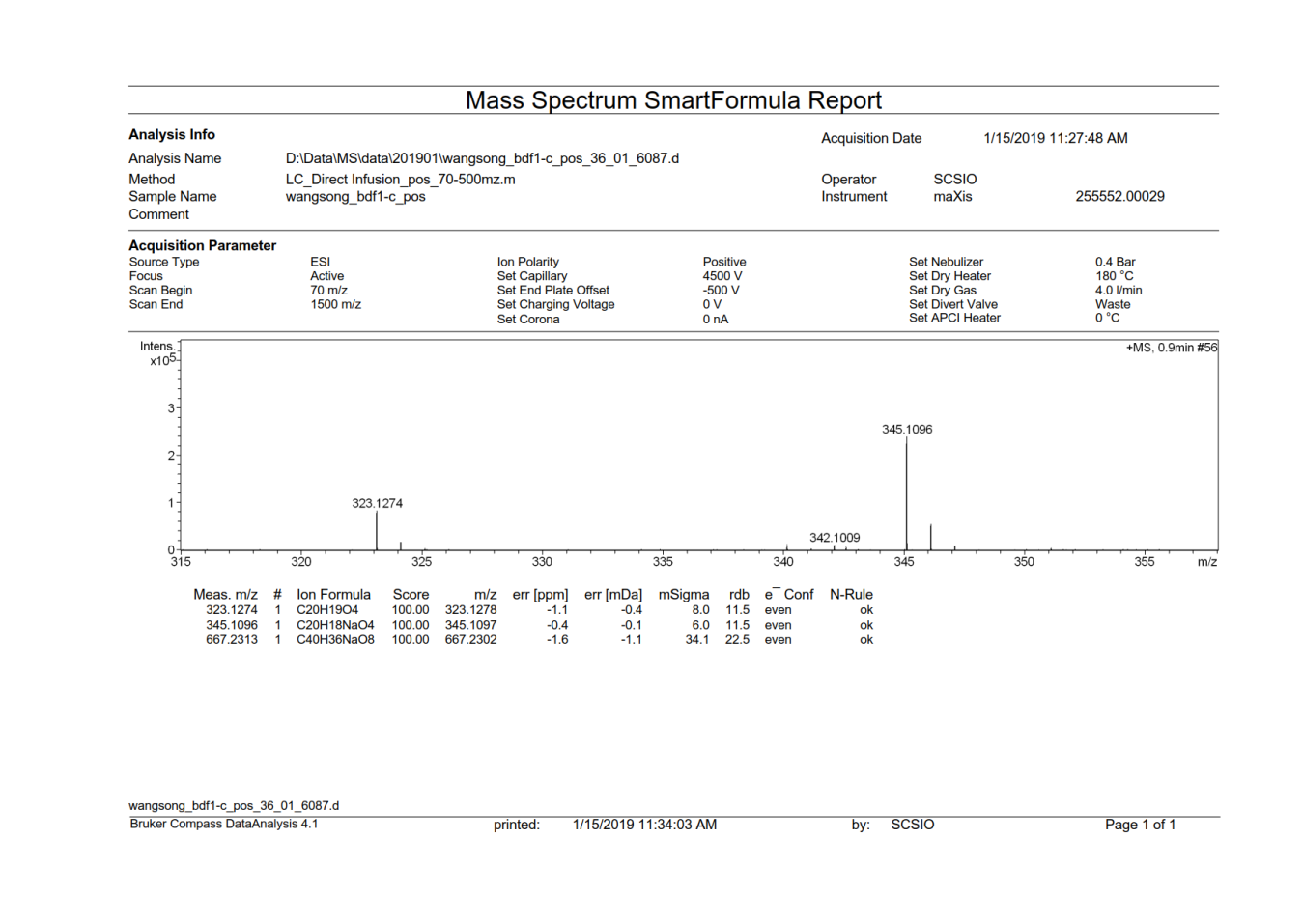


# Figure S21 HR-ESI-MS spectrum of 4″-Ddeoxyterphenyllin (4)


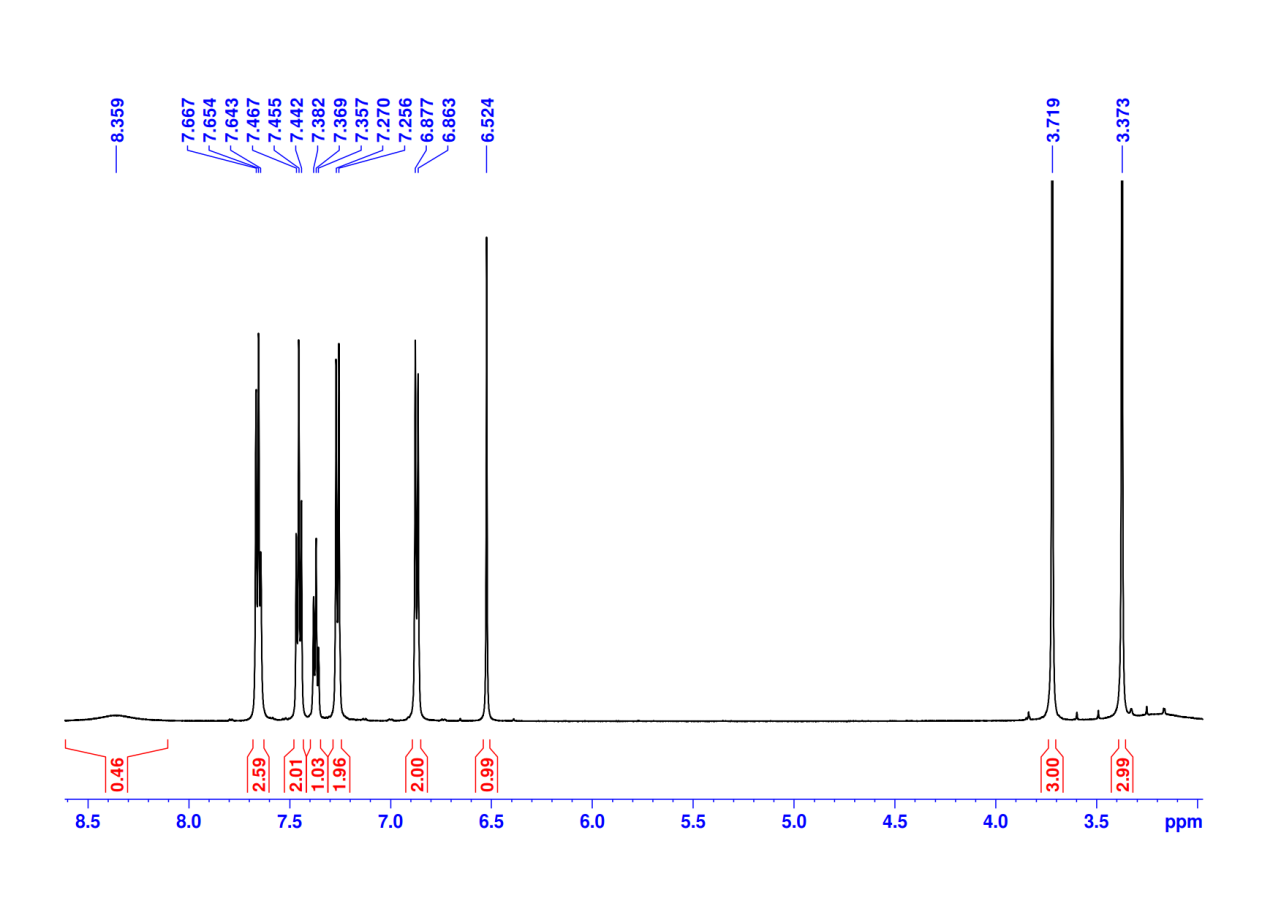


# Figure S22 ^1^H NMR spectrum of 4″-Ddeoxyterphenyllin (4) (Acetone-*d*_6_, 600 MHz)


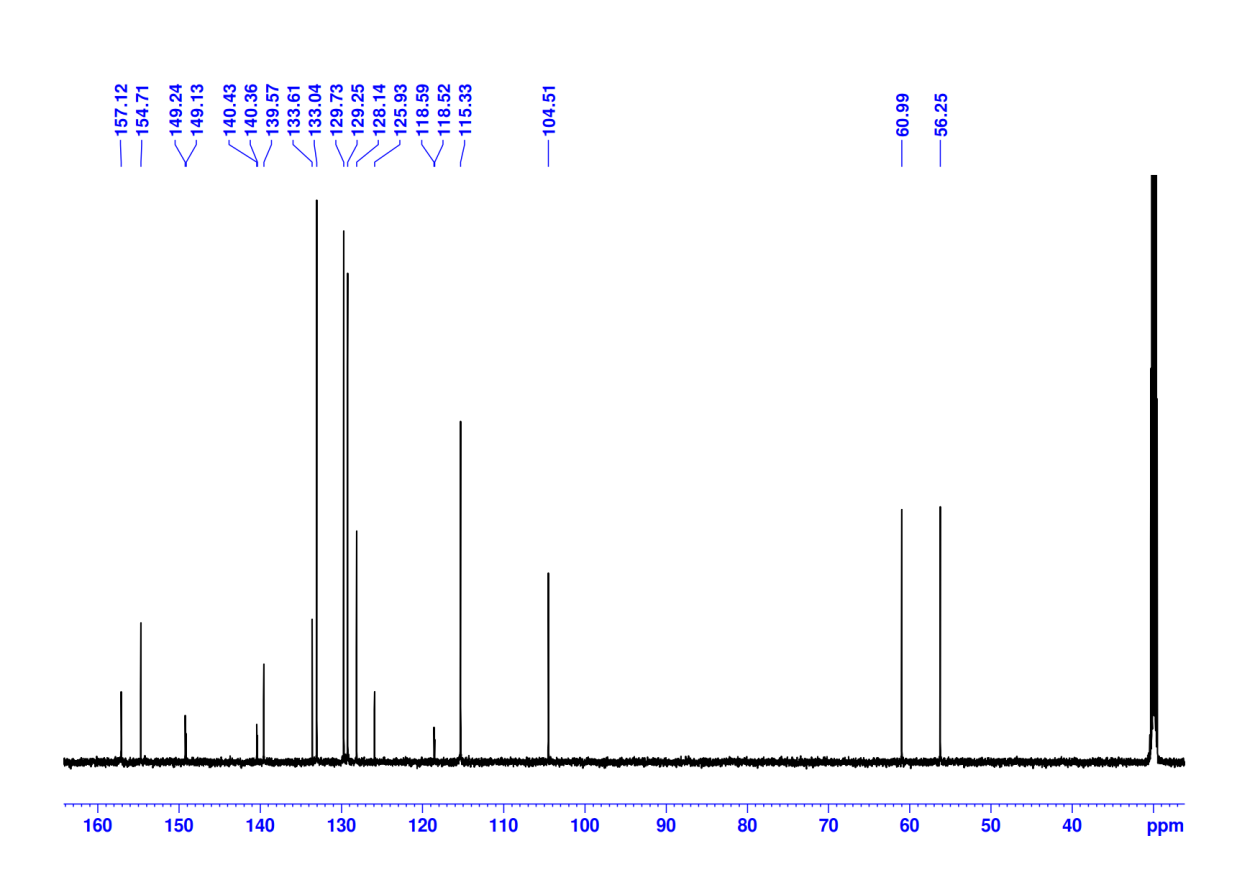


# Figure S23 ^13^C NMR spectrum of 4″-Ddeoxyterphenyllin (4) (Acetone-*d*_6_, 151 MHz)


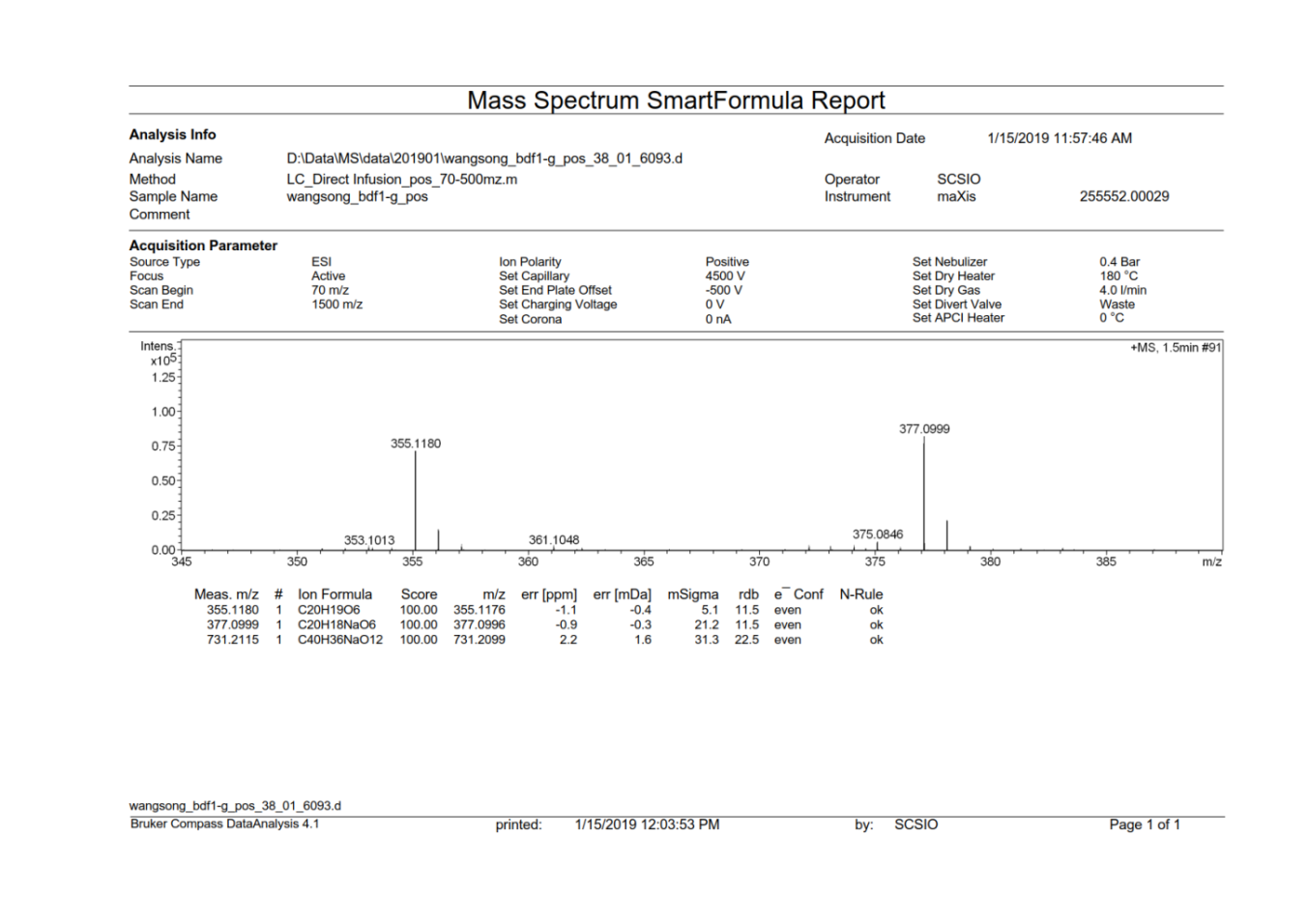


# Figure S24 HR-ESI-MS spectrum of 3''-Hydroxyterphenyllin (5)


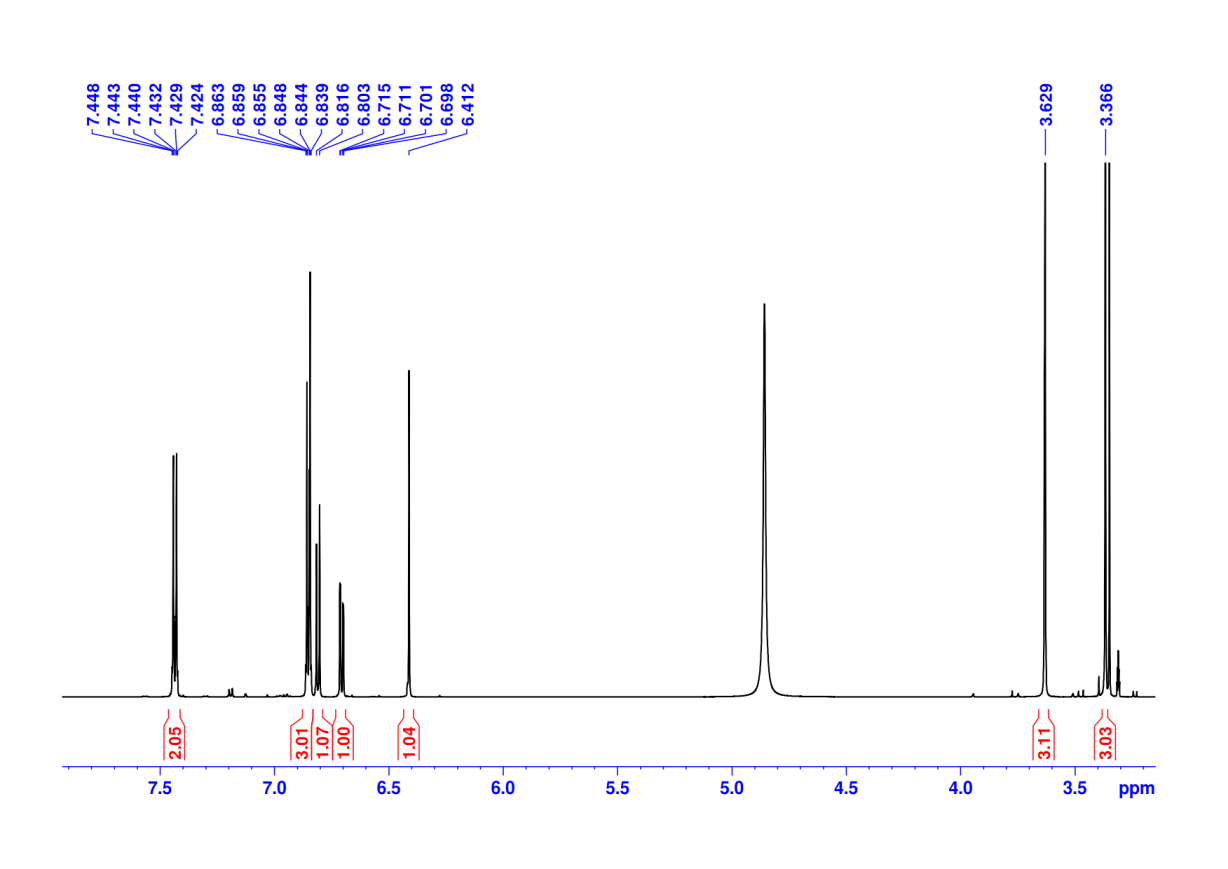


# Figure S25 ^1^H NMR spectrum of 3''-Hydroxyterphenyllin (5) (CD_3_OD, 600 MHz)


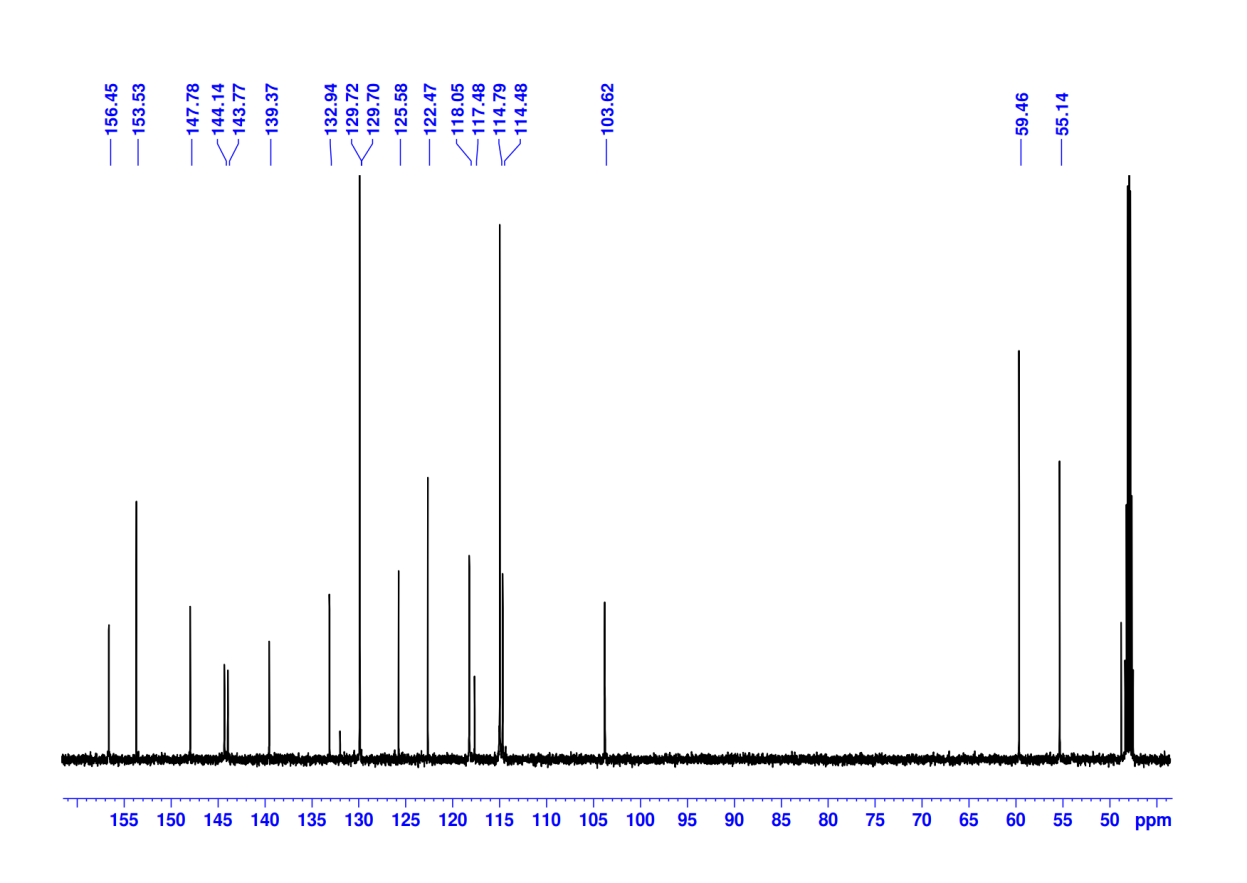


# Figure S26 ^13^C NMR spectrum of 3''-Hydroxyterphenyllin (5) (CD_3_OD, 151 MHz)


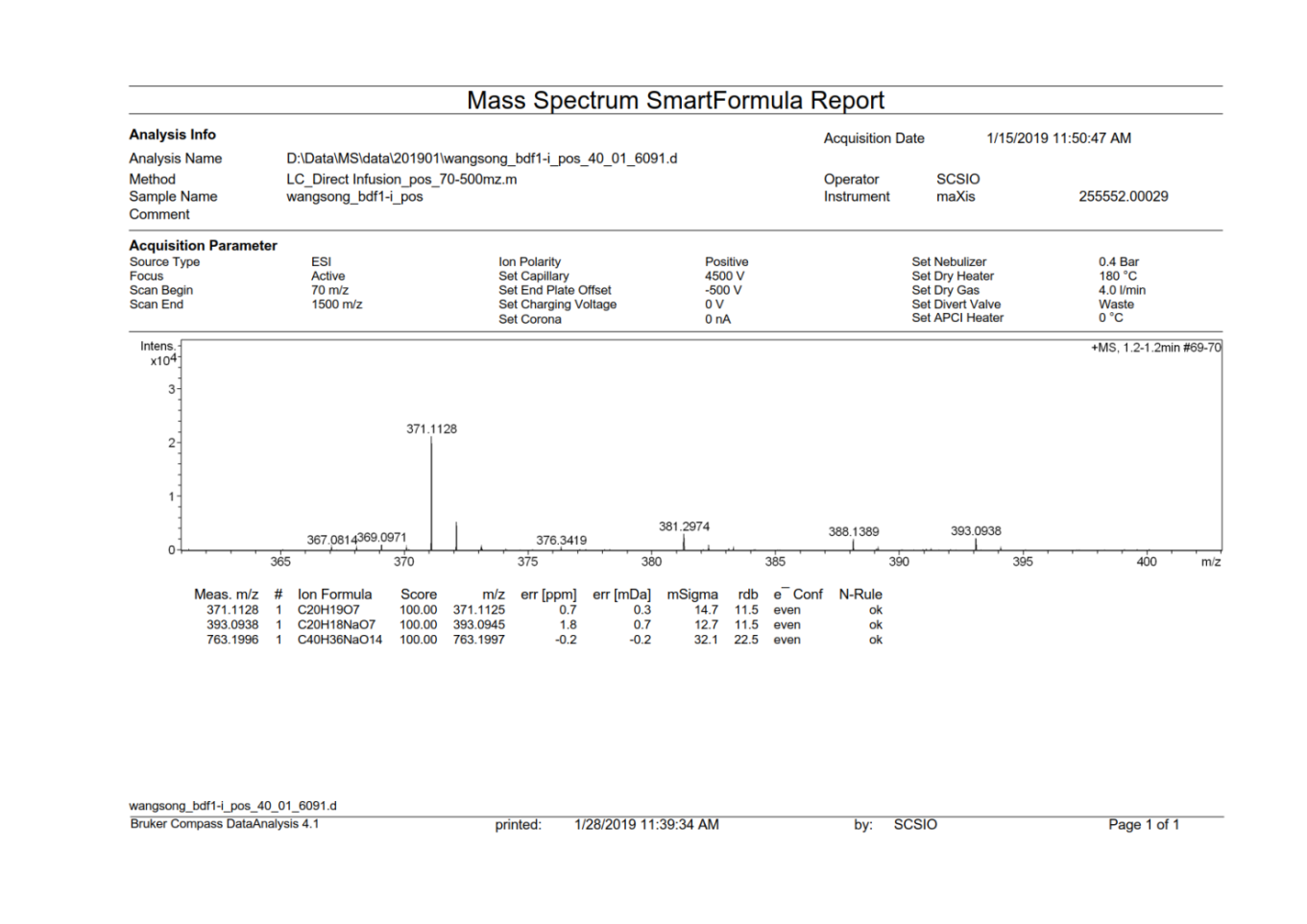


# Figure S27 HR-ESI-MS spectrum of 3, 3''-Dihydroxyterphenyllin (6)


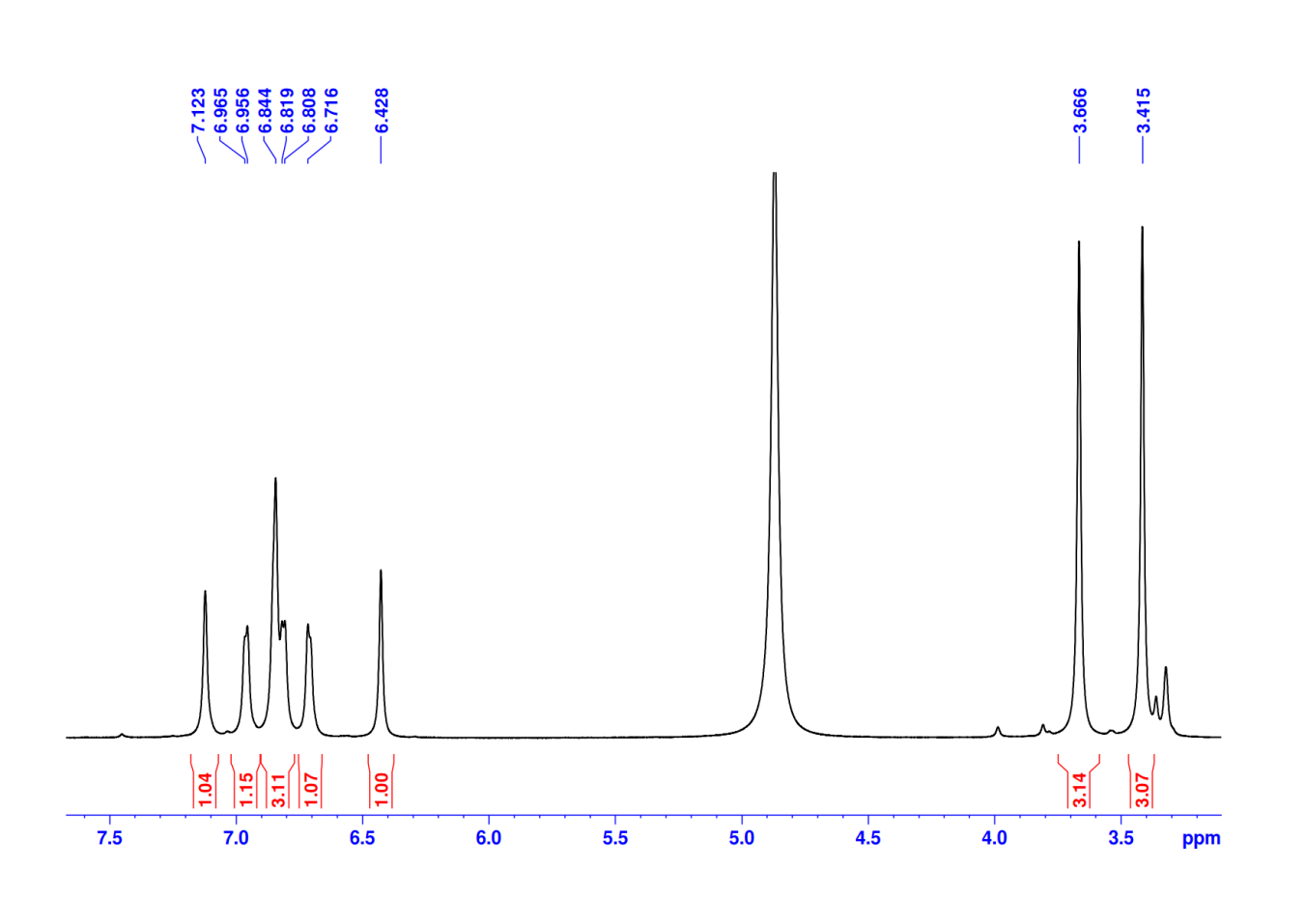


# Figure S28 ^1^H NMR spectrum of 3, 3''- Dihydroxyterphenyllin (6) (CD_3_OD, 600 MHz)


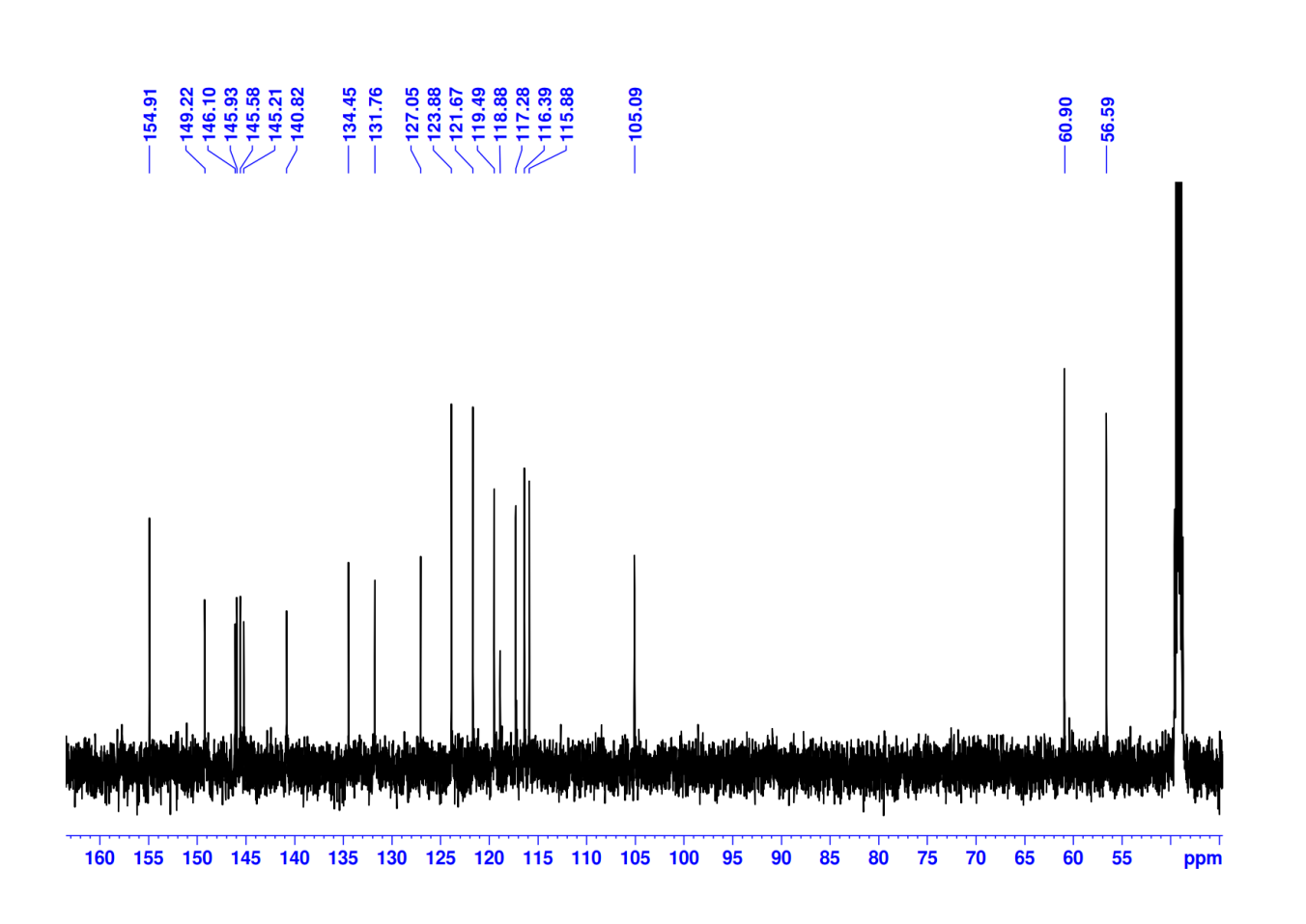


# Figure S29 ^13^C NMR spectrum of 3, 3''- Dihydroxyterphenyllin (6) (CD_3_OD, 151 MHz)


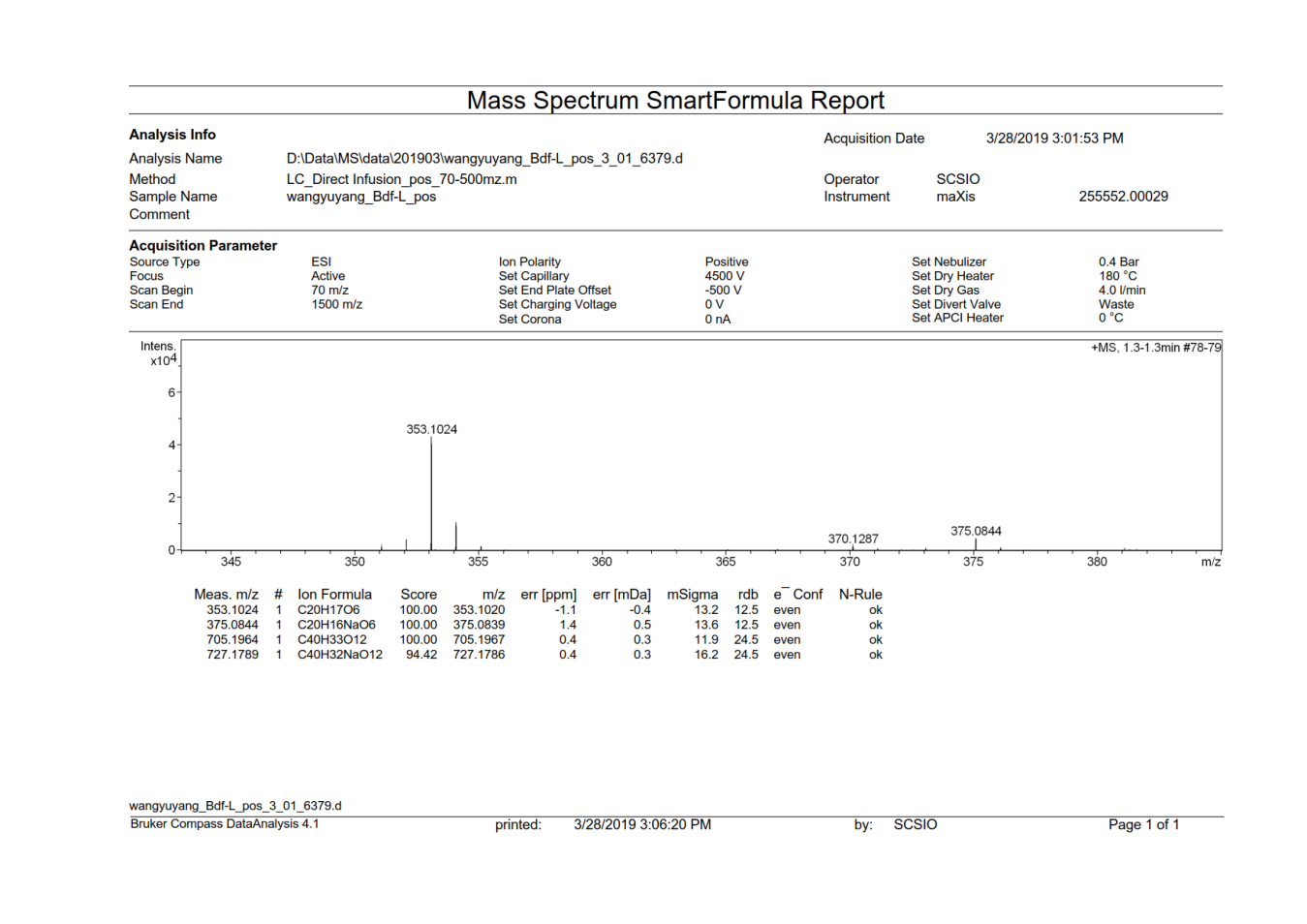


# Figure S30 HR-ESI-MS spectrum of Candidusin A (7)


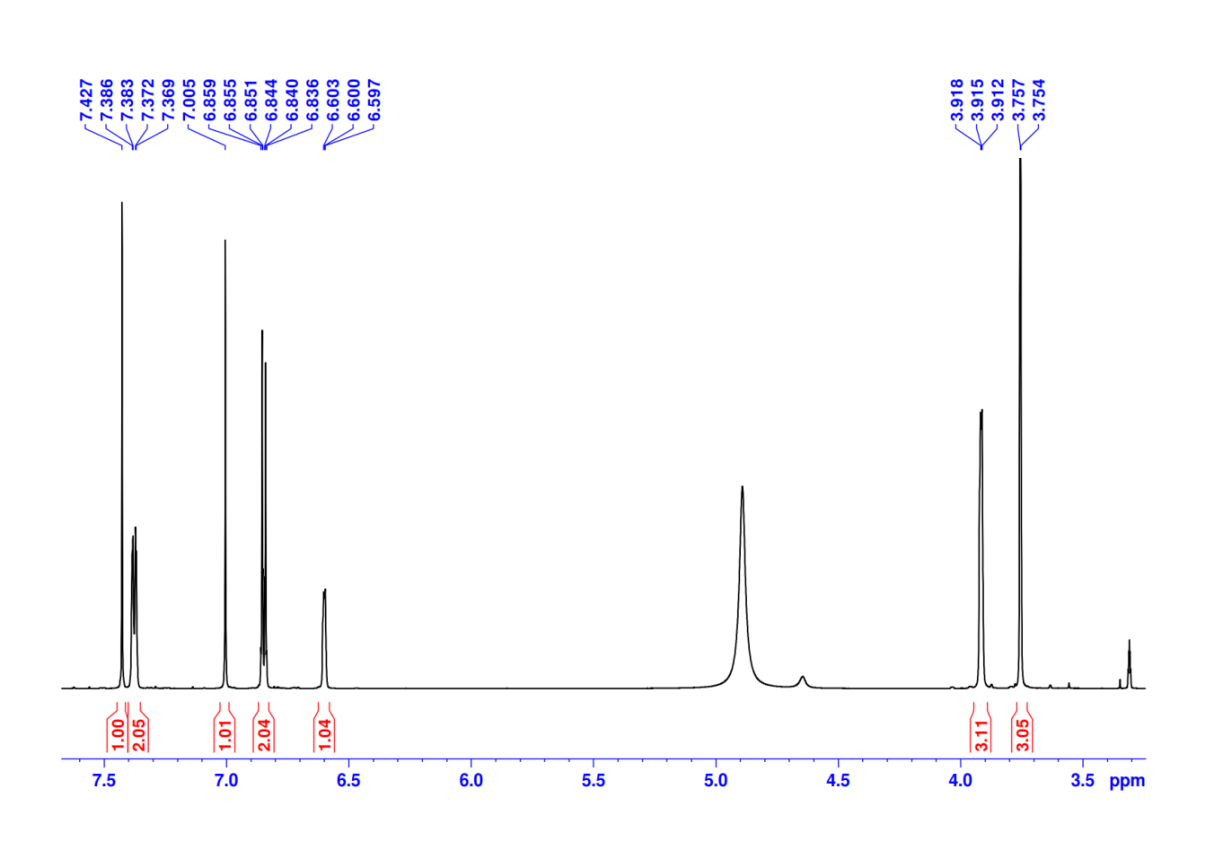


# Figure S31 ^1^H NMR spectrum of Candidusin A (7) (CD_3_OD, 600 MHz)


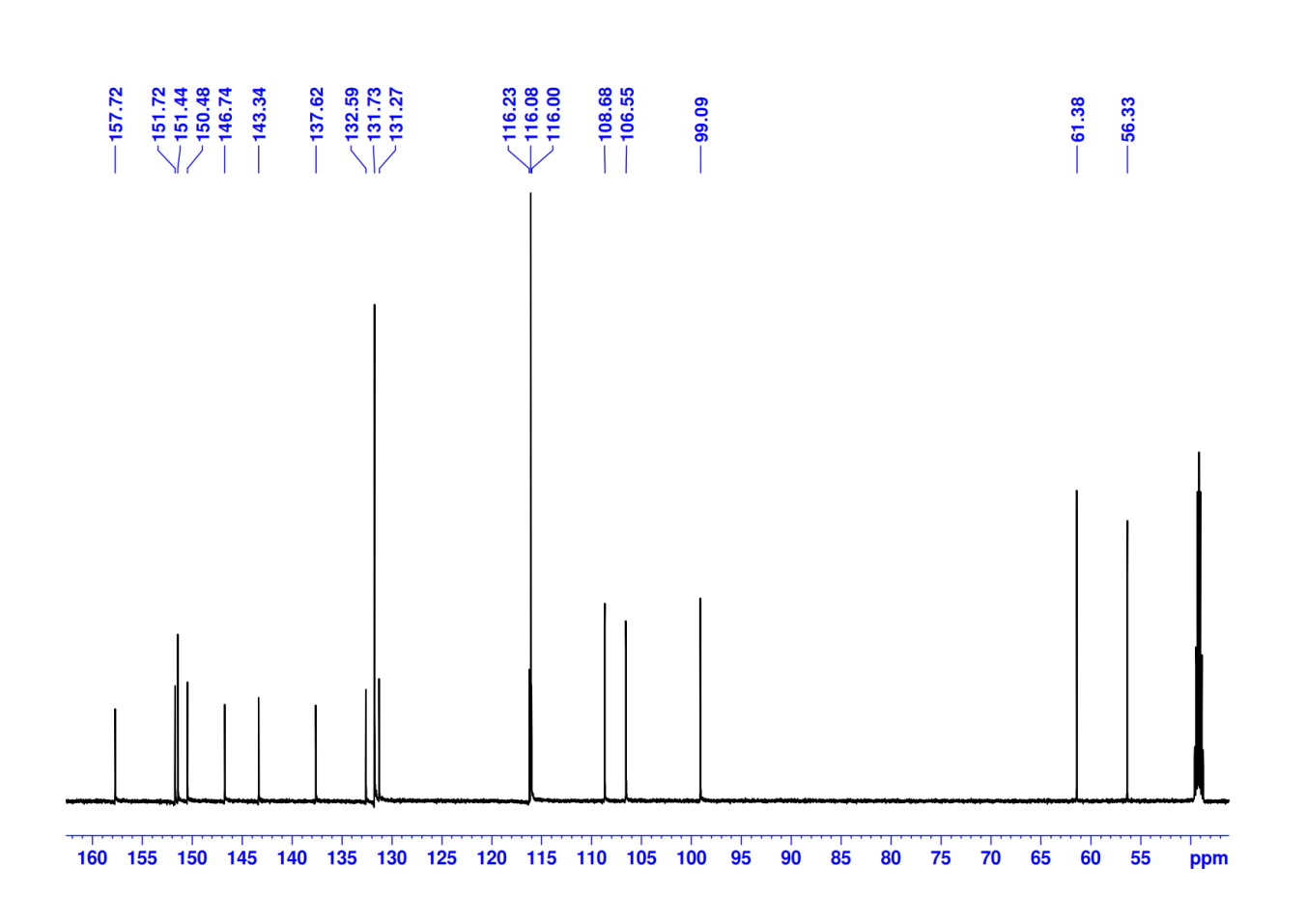


# Figure S32 ^13^C NMR spectrum of Candidusin A (7) (CD_3_OD, 151 MHz)


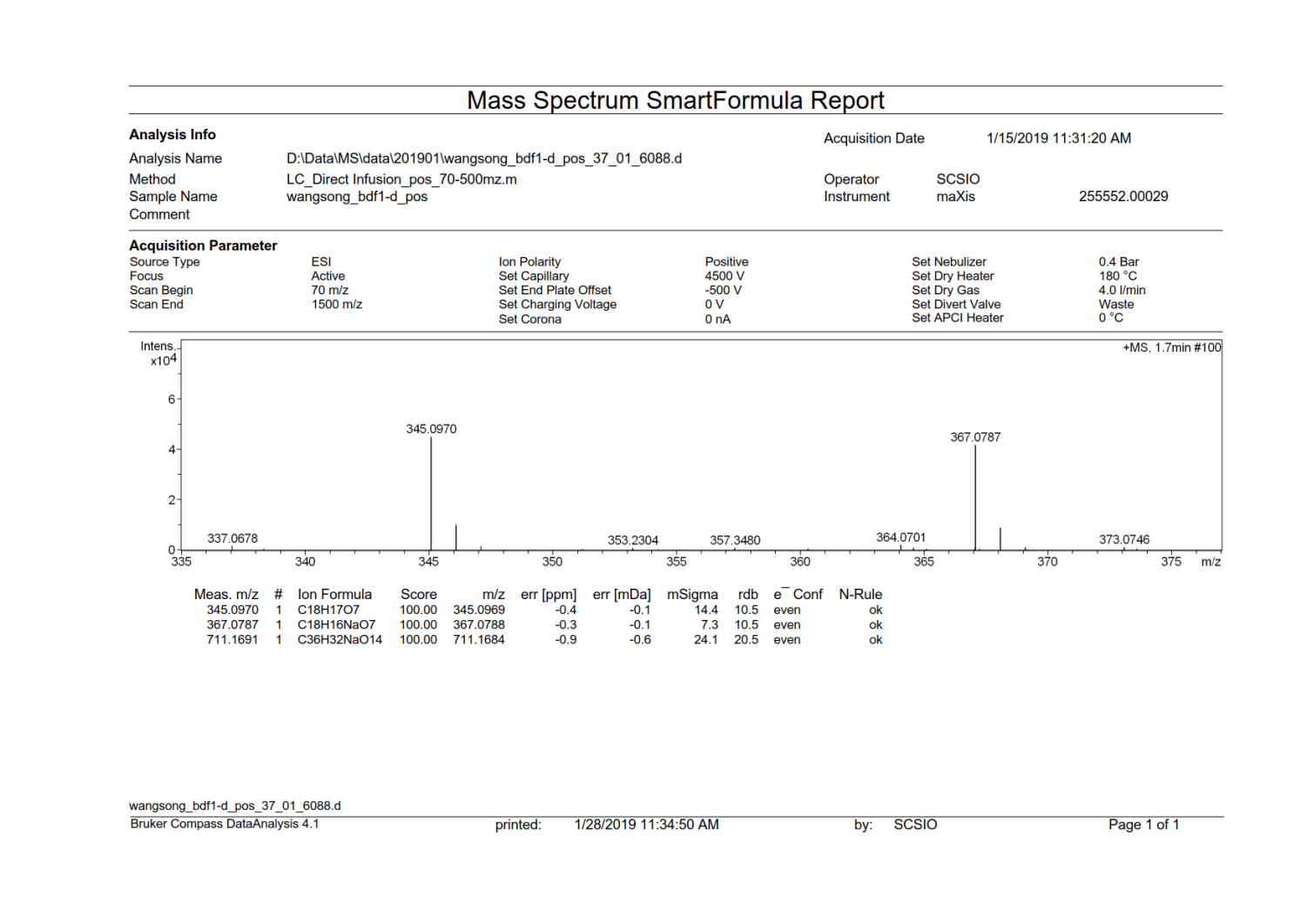


# Figure S33 HR-ESI-MS spectrum of Dechlorochlorflavonin (8)


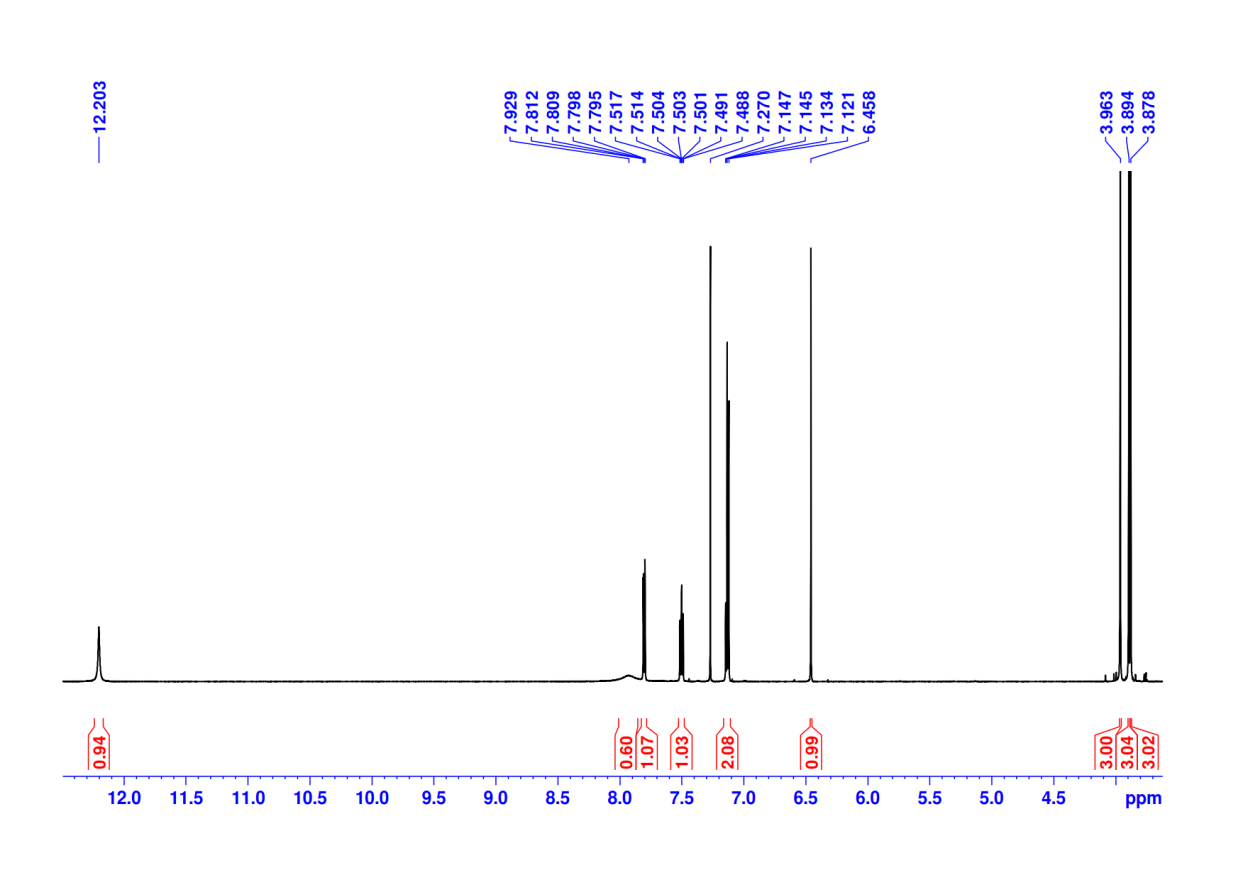


# Figure S34 ^1^H NMR spectrum of Dechlorochlorflavonin (8) (CDCl_3_, 600 MHz)


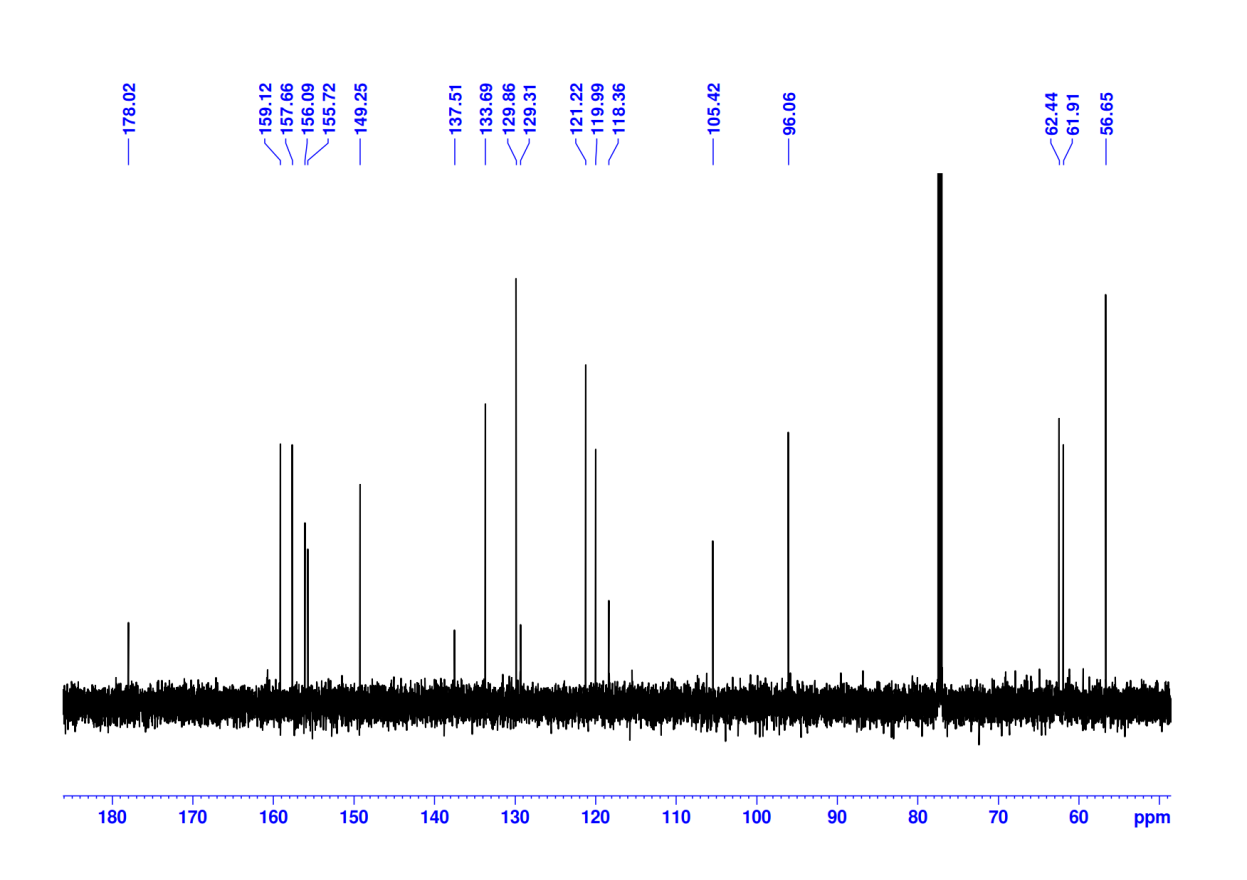


# Figure S35 ^13^C NMR spectrum of Dechlorochlorflavonin (8) (CDCl_3_, 151 MHz)


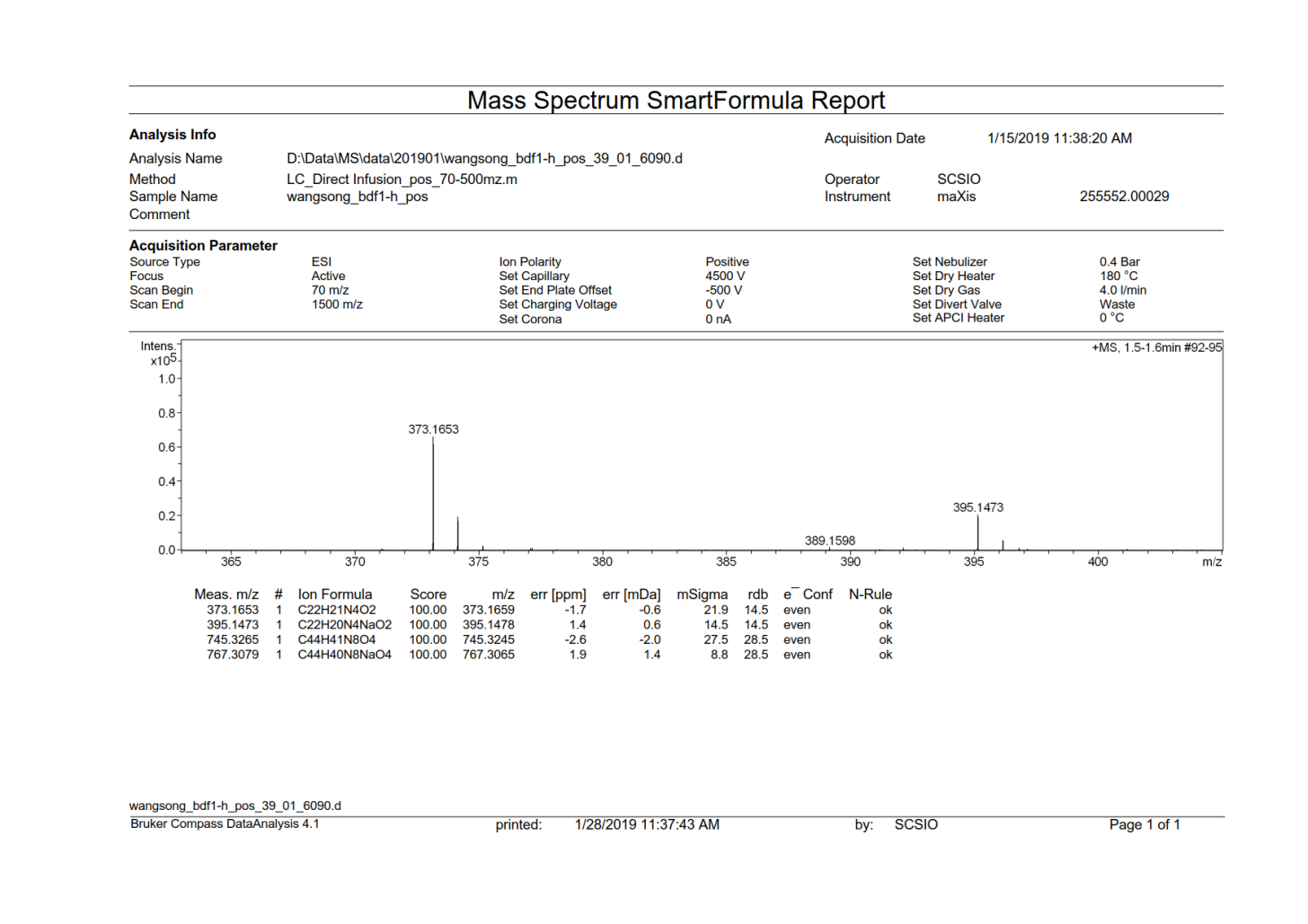


# Figure S36 HR-ESI-MS spectrum of Fellutanine A (9)


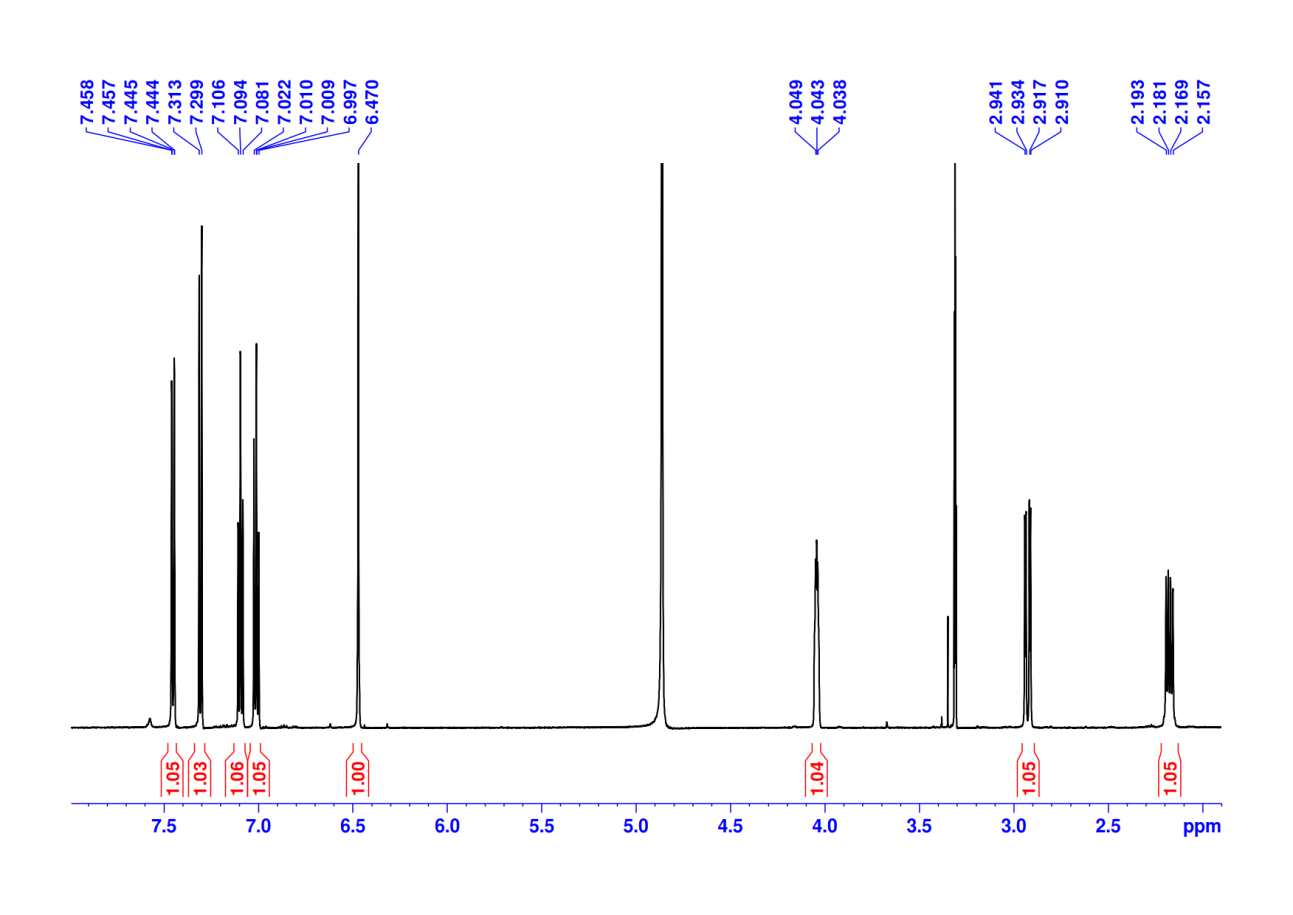


# Figure S37 ^1^H NMR spectrum of Fellutanine A (9) (CD_3_OD, 600 MHz)


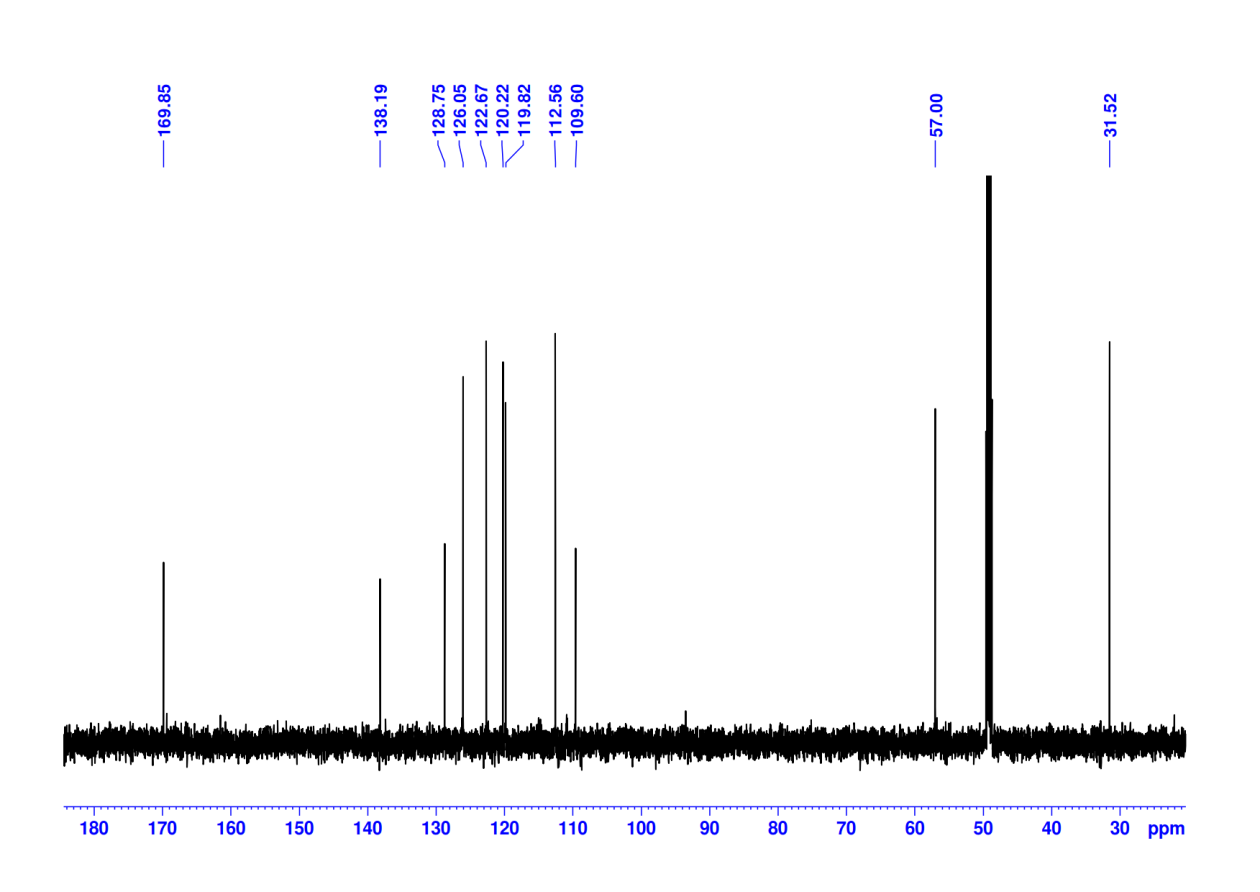


# Figure S38 ^13^C NMR spectrum of Fellutanine A (9) (CD_3_OD, 151 MHz)
