# Supplementary material for: A new p-terphenyl derivative from the insect-derived fungus Aspergillus candidus Bdf-2 and the synergistic effects of terphenyllin
Source: PeerJ. 2020 Jan 2;8:e8221. doi: 10.7717/peerj.8221 (PMC6942676; doi:10.7717/peerj.8221)
Supplement: Supplemental Information 3 [file peerj-08-8221-s004.docx]

Seq1 beta-tubulin gene (GenBank accession No. MN533958)

GGTAACCAAATCGGTGCTGCTTTCTGGTACGTCAAGATCAAGTGCAGGACGGACATGGCGGGGTCACGTGGAATGAGAGACTCCGTCCTGTCGCTGCCAACAAGGTCTCACTTCAATGACATTGATTCGCTGACAACCATGCAGGCAGACCATCTCCGGCGAGCACGGCCTTGACGGCTCCGGTGTGTAAGTATTGCCTGAACGCCCACGGGTATACCCGATTCGGGAATGGGAGAGCGTTGGGGAAGGGGATGTCGGTCCGTCTAACACTAAAGATAGGTACAATGGCACCTCCGACCTCCAGCTGGAGCGCATGAACGTCTACTTCAACGAGGTTCGTACTCGCGATCGTCTGCTCTGAAGAATATACACGACAGAATCCCTAATTCCCACCTCAGGCTAGCGGCAACAAGTATGTCCCCCGTGCCGTCCTCGTCGATCTGGAGCCCGGTACCATGGACGCCGTCCGTGCCGGTCCTTTCGGTCAGCTCTTCCGCCCCGACAACTTCGTCTTCGGCCAGTCCGGTGCTGGTAACAACTGGGCCAAGGGTCACTACACTGAGGGT

Seq2 RPB2 gene (GenBank accession No. MN533959)

AAAGGGCGTTAATGGGCGGTCCGCTGCTCGCCAACTTGTTCCGTGTTCTCTTCACTCGTGTGACGCGCGATCTTCAGCGATACGTGCAGCGCTGTGTGGAGACCAACCGTGAGATCTACTTGAACATCGGTATCAAGGCCAGCACCTTAACGGGAGGTCTGAAATATGCCCTCGCCACCGGTAACTGGGGTGAGCAGAAGAAGGCGGCTAGTTCCAAGGCCGGTGTATCGCAGGTGCTCAGTCGTTATACCTACGCCTCAACCCTGTCCCATTTGCGTCGGACCAACACCCCCATTGGTCGAGACGGTAAGATCGCCAAGCCCCGTCAACTGCACAACACGCACTGGGGCTTGGTCTGTCCTGCCGAGACACCTGAAGGTCAGGCTTGTGGTCTGGTTAAGAATTTGGCACTTATGTGTTACATCACTGTCGGTACACCTAGTGAGCCTATCATCGATTTCATGATTCAGCGGAACATGGAGGTTCTCGAGGAGTTCGAGCCCCAGGTCACACCGAACGCCACCAAGGTGTTTGTGAACGGCGTGTGGGTCGGAATTCACCGCGACCCGGCGCATTTGGTCAATACGATGCAGTCGCTGCGCCGGCGGAACATGATCTCGCACGAGGTCAGCTTGATCCGGGACATCCGTGAACGGGAGTTCAAGATCTTCACCGATGCCGGGCGCGTGTGCCGTCCGTTGTTCGTCATCGACAACGATCCGAAGAGCGAGAATTGCGGATCGCTGGTTCTCAACAAGGAACACATTCGCAAGCTCGAGCAGGACCGAGAACTGCCGCCGGATCTGGACCCGGAAGAGCGCCGAGAACGCTATTTCGGATGGGACGGTCTGGTGAAGTCGGGAGCGGTCGAATACGTGGACGCGGAGGAAGAAGAAACGATCATGATCGTCATGACCCCCGAAGACCTGGAGATCTCCAAGCAGCTCCAGGCCGGCTATGCACTCCCCGAGGAGGAGCTCCACGACCCGAACAAGCGTGTGCGCTCCATTCTTAGTCAGCGGGCTCACACTTGGACACATTGTGAGATCCATCCTAGTATGATTCTCGGGGTGTGCGCCAGTATCATTCCGTTCCCCGATCATAACCAGTCGCCTCGACCTTCAAGGGCCTC

Seq3 Large Subunit Ribosomal RNA gene (GenBank accession No. MN533960)

GGGGGACCCGAATGGGTTCGATTAGTCTTTCGCCCCTATACCCAAATTCGACGATCGATTTGCACGTCAGAACCGCTGCGAGCCTCCACCAGAGTTTCCTCTGGCTTCGCCCTATTCAGGCATAGTTCACCATCTTTCGGGTCCCCACAGCTACGCTCCTACTCAAATCCATCCGAAGACATCAGGATCGGTCGATGGTGCGCCCCTCAGGGGGCTCCCACCTCCGTTCGCTTTCACTGCGCGCACGGGTTTGACACCCGAACACTCGCGTAGATGTTAGACTCCTTGGTCCGTGTTTCAAGACGGGTCGTTTACGACCATTATGCCAGCGTCCGTGCCGAAGCGCGTTCCTCGGTCCAGGCTGGCCGCACTGCACCCCCGGCTATAAGGCGCCCCGAGAGGCGCTACATTCCGGGAGCCGCTGACCGGCCGCCCAAACCGACGCTGGCCCGCCCACGGGGAAGTACACCGGCACGAATGCCGGCTGAACCCCGCGGGCGAGTCTGGTCGCAAGCGCTTCCCTTTCAACAATTTCACGTGCTGTTTAACTCTCTTTTCAAAGTGCTTTTCATCTTTCGATCACTCTACTTGTGCGCTATCGGTCTCCGGCCAATATTTAGCTTTAGATGAAAATTTACCACCCATTTAGAGCTGCATTCCCAAACAACTCGACTCGTCGAAGGAGCTTCACACGGGCGCGGACACCCCCATCCCAGACGGGGATTCTCACCCTCTCTGACAGCCCCGTTCCAGGGCACTTAGACAGGGGGCCGCACCCGAAGCATCCTCTGCAAATTACAATGCGGACCCCCGAAGGAGCCAGCTTTCAAATTTGAGCTCTTTGCCGCTTCACTCGCAGTTACTGAGGCAATCCCGGTTGGTTTCTTTTCCTCCGCTTATTGATATGCTTAATTCAGCGGGTACAATAAAATGCT

Seq4 ITS gene (GenBank accession No. MH681592)

TCCTCCGCTTATTGATATGCTTAAGTTCAGCGGGTATCCCTACCTGATCCGAGGTCAACCTGTAAAAAATGGTTGGGTTGGTCGGCTGGCGCCGGCCGGGCCTGCAGAGCGGGTGACAAAGCCCCATACGCTCGAGGACCGGACGCGGTGCCGCCGCTGCCTTTCGGGCCCGTCCCCGGGGGTACCGGGGACGGGGCCCAACACACAAGCCGTGCTTGAGGGCAGCAATGACGCTCGGACAGGCATGCCCCCCGGAATACCAGGGGGCGCAATGTGCGTTCAAAGACTCGATGATTCACTGAATTCTGCAATTCACATTAGTTATCGCATTTCGCTGCGTTCTTCATCGATGCCGGAACCAAGAGATCCATTGTTGAAAGTTTTGACTGATTGGTAACAATCGACTCAGACTGCACTTTTCAGACAGTGTTCGTGTTGGGGTCTTCGGCGGGCGCGGGCCCGGGGACGCGAGGTCCCCCGGCGGCCGTGAGGCGGGCCCGCCGAAGCAACAGGGTACGGTATACACGGGTGGGAGGTTGGGCTTCAGAGAAACCCTCACTCGGTAATGATCCTTCCGCAGGTTCACCTACGGAAACCTTGTTACGACTTTTACTTCC
